# Transcriptome Analysis Identifies Two Ethylene Response Factors That Regulate Proanthocyanidin Biosynthesis During *Malus* Crabapple Fruit Development

**DOI:** 10.3389/fpls.2020.00076

**Published:** 2020-02-26

**Authors:** Hua Li, Mingzheng Han, Lujia Yu, Sifan Wang, Jie Zhang, Ji Tian, Yuncong Yao

**Affiliations:** ^1^ Beijing Advanced Innovation Center for Tree Breeding by Molecular Design, Beijing University of Agriculture, Beijing, China; ^2^ Department of Plant Science and Technology, Beijing University of Agriculture, Beijing, China

**Keywords:** *Malus* crabapple, proanthocyanidins, RNA-seq, ethylene, ethylene response factors

## Abstract

Proanthocyanidins (PAs) are a class of flavonoid compounds in plants that play many important roles in pest and disease resistance and are beneficial components of the human diet. The crabapple (*Malus*) provides an excellent model to study PA biosynthesis and metabolism; therefore, to gain insights into the PA regulatory network in *Malus* plants, we performed RNA-seq profiling of fruits of the ‘Flame’ cultivar at five sequential developmental stages. KEGG (*Kyoto Encyclopedia of Genes and Genomes*) enrichment analysis showed that differentially expressed genes (DEGs) related to the functional category ‘plant hormone signal transduction’ were significantly enriched during fruit development. Further analysis showed that ethylene signal transduction pathway genes or response genes, such as ERS (ethylene response sensor), EIN3 (ETHYLENE INSENSITIVE 3) and ERFs (ethylene response factors), may play an important role in the regulatory network of PA biosynthesis. Additionally, 12 DEGs, including 10 ERFs, 1 MYB, and 1 bHLH transcription factor, associated with PA biosynthesis were identified using WGCNA. The expression patterns of these genes correlated with PA accumulation trends and transcriptome data from qRT-PCR analysis. The expression of *RAP2-4* (RELATED TO APETALA 2-4) and *RAV1* (related to ABI3/VP1), which belong to the ERF transcription factor family, showed the greatest correlations with PAs accumulation among the 12 identified TFs. *Agrobacterium* mediated-transient overexpression of the *RAP2-4* led to an increase in PA abundance in crabapple leaves and apple fruits, and the opposite results were observed in *RAV1*-overexpressed crabapple leaves and apple fruits. Moreover, a yeast one-hybrid assay showed that RAP2-4 and RAV1 specifically bound the promoters of the PA biosynthetic genes *McLAR1* and *McANR2*, respectively. These results indicate that RAP2-4 act as an inducer and RAV1 act as a repressor of PA biosynthesis by regulating the expression of the PA biosynthetic genes *McLAR1* and *McANR2*. Taken together, we identified two potential regulators of PA biosynthesis and provide new insights into the ethylene-PA regulatory network.

## Introduction

Flavonoids compose a major class of plant polyphenolic compounds and can be divided into three categories: anthocyanins, proanthocyanidins (PAs) and flavonols (Williams and Grayer, 2004). PAs are formed by the condensation of flavan-3-ol monomeric units (catechin and epicatechin) and are also called condensed tannins. PAs are known to be involved in protection against UV radiation and defense against microbial pathogens and pest attacks (Carini et al., 2000; Li M. et al., 2016), and they have multiple health benefits in the human diet as a consequence of their antioxidant activities (Yun et al., 2011; Ma et al., 2017).

Leucoanthocyanidin reductase (LAR) and anthocyanidin reductase (ANR) are located at the branch of the common core flavonoid pathway (Tanner et al., 2003; Bogs et al., 2005), are mainly responsible for the biosynthesis of PAs *via* a multistep enzymatic reaction and have been studied in many plant species (Xie et al., 2003; Xie et al., 2004). In apple, the transcript levels of the *LAR* and *ANR* genes were significantly correlated with the contents of catechin and epicatechin, respectively, which suggests that they play important roles in PA synthesis (Liao et al., 2015). Moreover, two LAR genes, *MrLAR1* and *MrLAR2*, and two ANR genes, *MrANR1* and *MrANR2*, have been identified in crabapple. Overexpressing these four genes in tobacco leaves (*Nicotiana tabacum*) increased the PA content, and silencing them in crabapple plants inhibited the accumulation of PAs (Li et al., 2019).

Several studies have reported that genes involved in flavonoid biosynthesis are regulated by transcription factors of the R2R3-MYB, bHLH (basic helix-loop-helix) and conserved WD repeat families. For example, in apple, the anthocyanin pathway is controlled by MdMYB1, MdMYB10, and MdMYBA. Overexpressing these three TFs in apples can activate the expression of anthocyanin biosynthetic genes and significantly promote anthocyanin accumulation in plants (Ban et al., 2007; Espley et al., 2007; Takos et al., 2006). A recent study suggested that a paralog of MdMYB10, MdMYB110a, regulates anthocyanin accumulation in red-fleshed apples (Chagné et al., 2013). The low-temperature-induced MdbHLH3 protein interacts with MdMYB1 and promotes anthocyanin accumulation by activating the expression of MdMYB1 and anthocyanin biosynthetic genes in apple (Xie et al., 2012). Similar results were also found in other plants, such as Arabidopsis (*Arabidopsis thaliana*), alfalfa (*Medicago truncatula*), and strawberry (*Fragaria* × *ananassa*) (Gonzalez et al., 2008; Li P. et al., 2016; Medina-Puche et al., 2014). Moreover, several studies have shown that MYB and bHLH TFs are also involved in PA biosynthesis (Espley et al., 2007; Hichri et al., 2011; Montefiori et al., 2015; Lai et al., 2016). In apple (*Malus domestica*), overexpressing MdMYB9 or MdMYB11 increased the contents of both anthocyanins and PAs in apple calli (Wang et al., 2017). Additionally, both MYB proteins interact with MdbHLH3 and bind to the promoters of *MdANS*, *MdANR*, and *MdLAR* (An et al., 2015). Recently, MdMYBPA1, a PA1-type MYB TF, was cloned from red-fleshed apple; overexpressing MdMYBPA1 could promote PA accumulation in apple calli by binding the promoters of genes in the PA and anthocyanin biosynthetic pathways (Wang N. et al., 2018). In crabapple, McMYB12a and McMYB12b coordinately regulate PA biosynthesis by binding to the promoters of PA biosynthetic genes (Tian et al., 2016). Moreover, several MYB transcription factors, such as VvMYBC2-L1, VvMYBPA1, VvMYBPA2, and VvMYBPAR, have been shown to specifically regulate the PA biosynthetic pathway by significantly activating enzymes in the flavonoid pathway in grape (*Vitis vinifera*) (Huang et al., 2014; Koyama et al., 2014).

There are also a growing number of reports that other regulatory genes are involved in regulating PA biosynthesis (Sagasser et al., 2002; Amato et al., 2016; Gonzalez et al., 2016); for example, a BTB protein, MdBT2, plays a negative role in the biosynthesis of anthocyanins and proanthocyanidins. MdBT2 interacts with MdMYB9 and negatively regulates the abundance of MdMYB9 protein *via* the 26S proteasome system (An et al., 2018a). The ethylene response factor MdERF1B has been shown to interact with MdMYB9, MdMYB1, and MdMYB11 to regulate anthocyanin and proanthocyanidin biosynthesis (Zhang et al., 2018). Thus, we speculate that there may be many different transcription factor families involved in PA biosynthesis in crabapple. MdNAC52 (NAM, ATAF1/2, CUC2) participates in the regulation of PA biosynthesis by regulating the expression of MdMYB9 and MdMYB11. Additionally, MdNAC52 can directly bind the promoter of *MdLAR* to control its expression and promote PA synthesis (Sun et al., 2019).

Crabapple (*Malus*) belongs to the *Malus Mill* family of Rosaceae and is represented by a large germplasm collection (Tian et al., 2011); its fruits produce abundant anthocyanins, flavonols, PAs, and phlorizin compounds. Procyanidin B1, procyanidin B2, epicatechin, and catechin are the main PA compounds in crabapple fruits (Tian et al., 2017; Li et al., 2019). This makes it a valuable model for studying the molecular mechanisms of PA biosynthesis. In the current study, we performed RNA-seq analyses on the fruit of the ‘Flame’ crabapple cultivar at five different developmental stages to identify candidate regulators of PA biosynthesis. Furthermore, we conducted an unbiased network analysis to identify genes that are coexpressed with those known to be involved in PA accumulation. Based on the results of qRT-PCR analysis, transient infection, 4-dimethylaminocinnamaldehyde (DMACA) staining and yeast one hybrid (Y1H), we characterized the functions of two transcription factors involved in PA biosynthesis during fruit development.

## Materials and Methods

### Plant Materials

In this study, *Malus* spp. ‘Flame’, a green-fruited cultivar, was used. Eight-year-old trees were grafted onto *Malus hupehensis* and planted at the Crabapple Germplasm Resources Nursery at the Beijing University of Agriculture (40.l°N, 116.6°E). Three trees showing similar growth were used, and fruit samples were collected from annual branches growing at the edge in the southeast direction. The fruits were collected 20, 40, 60, 80, and 100 days after budding (S1-S5, ‘S’ represents ‘stage’). All flesh samples were frozen in liquid nitrogen upon collection and stored at −80°C prior to high-pressure liquid chromatography (HPLC) analysis or RNA extraction.

‘Flame’ tissue culture plants were harvested from one-year-old branches before spring bud germination, and the culturing conditions were as previously described (Tian et al., 2017).

### RNA Quantification and Quality Analysis

RNA degradation and contamination were visualized on 1% agarose gels. RNA purity was confirmed using a Nano Photometer^®^ spectrophotometer (IMPLEN, CA, USA). RNA concentration was measured using a Qubit^®^ RNA Assay Kit in a Qubit^®^ 2.0 fluorometer (Life Technologies, CA, USA). RNA integrity was assessed using an RNA Nano 6000 Assay Kit and a Bioanalyzer 2100 system (Agilent Technologies, CA, USA) (Yang et al., 2018).

### RNA-Seq Library Preparation

A total of 3 µg of RNA per sample was used as input material for the RNA sample preparation. Sequencing libraries were generated using an NEBNext^®^ Ultra™ RNA Library Prep Kit for Illumina^®^ (NEB, USA) following the manufacturer’s recommendations, and index codes were added to label each sample. To preferentially select the 150 to 200 bp cDNA fragments, an AMPure XP system was used to purify the library fragments. High-fidelity DNA polymerase, Universal PCR primers and index (X) primers were used in the PCRs. An Agilent Bioanalyzer 2100 system was used to assess the quality of the library.

### Read Mapping to the Reference Genome and Quantification of Gene Expression

An index of the reference genome was built using Bowtie v2.2.3, and paired-end clean reads were aligned to the apple (*Malus domestica*) reference genome using TopHat v2.0.12 (Trapnell et al., 2009; Riccardo et al., 2010). HTSeq v0.6.1 (https://pypi.python.org/pypi/HTSeq) was used to count the read numbers mapped to each gene (Anders et al., 2015).

### Differential Expression Analysis

Differential gene expression analysis of the five groups (three biological replicates per group) was performed using the DESeq R software package (1.18.0) (http://www.bioconductor.org/packages/release/bioc/html/DESeq.html) (Benjamini and Hochberg, 1995). The resulting *P*-values were adjusted using the Benjamini and Hochberg approach for determining the false discovery rate (Benjamini and Hochberg, 1995). Genes with an adjusted *P*-values < 0.05 found by DESeq were considered differentially expressed (Anders and Huber, 2010).

### Gene Ontology (GO) and *Kyoto Encyclopedia of Genes and Genomes* (KEGG) Enrichment Analysis of Differentially Expressed Genes (DEGs)

Blast2GO software was used to identify enriched GO terms. GO terms with corrected *P* < 0.05 were considered significantly enriched for DEGs (Conesa et al., 2005). KOBAS software was used to test the statistical enrichment of DEGs in the KEGG pathways (http://www.genome.jp/kegg/) (Mao et al., 2005).

### Identification of Coexpression Modules and Visualization of Hub Genes

The R WGCNA package was used to identify modules of highly correlated genes based on fragments per kilobase of transcript per million mapped reads (FPKM) data (Zhang and Horvath, 2005). The WGCNA analysis was performed according to established methods (Zhan et al., 2015). Genes with the highest degree of connectivity within a module are referred to as intramodular hub genes (Langfelder and Horvath, 2008). The gene annotation information was taken from the KOBAS 2.0 annotation results.

### HPLC and Quantitative Real Time (qRT)-PCR Analysis

HPLC and qRT-PCR analyses were performed according to previously published methods (Tian et al., 2011). Frozen samples (approximately 0.8–1.0 g fresh weight) were extracted with 10 mL extraction solution (methanol: water: formic acid: trifluoroacetic acid = 70: 27: 2: 1) at 4°C in the dark for 72 h. The supernatant was passed through filter paper and then through a 0.22-μm Millipore™ filter (Billerica, MA, USA). Trifluoroacetic acid: formic acid: water (0.1: 2: 97.9) was used as mobile phase A, and trifluoroacetic acid: formic acid: acetonitrile: water (0.1: 2: 48: 49.9) was used as mobile phase B for HPLC analysis. The gradients used were as follows: 0 min, 30% B; 10 min, 40% B; 50 min, 55% B; 70 min, 60% B; 30 min, 80% B. Detection was performed at 520 nm for anthocyanins and at 280 nm for PAs (Revilla and Ryan, 2000). All samples analyzed consisted of three biological triplicates.

The expression levels of related genes were analyzed using qRT-PCR and SYBR Green qPCR Mix (TaKaRa, Ohtsu, Japan) with a Bio-Rad CFX96 Real-Time PCR system (BIO-RAD, USA) according to the manufacturers’ instructions. The PCR primers were designed using NCBI Primer BLAST (https://www.ncbi.nlm.nih.gov/tools/primer-blast/) and are listed in **Table S1**. qRT-PCR analysis was carried out in a total volume of 20 μl containing 9 μl of 2×SYBR Green qPCR Mix (TaKaRa, Ohtsu, Japan), specific primers at 0.1 μM each, and 100 ng of template cDNA. The reaction mixtures were heated to 95°C for 30 s, followed by 39 cycles at 95°C for 10 s, 50 to 59°C for 15 s, and 72°C for 30 s. A melting curve was generated for each sample at the end of each run to ensure the purity of the amplified products. The transcript levels were normalized using the *Malus 18S ribosomal RNA* gene (GenBank ID DQ341382, for crabapple) as the internal control and calculated using the 2^ ^(−ΔΔCt)^ analysis method. All samples analyzed consisted of three biological replicates extracted from three different batches of fruits.

### DMACA Staining and Determination of PA Content

PA accumulation in crabapple leaves was visualized *via* infiltration with DMACA stain. Samples were stained *via* incubation overnight with DMACA solution (0.2% DMACA w/v in methanol: 6 M HCL, v/v = 1:1). The PA content was determined as previously described (Wang et al., 2017).

### Transient Expression Assays in Crabapple Plantlets and Apple Fruits

Full-length RAP2-4 (MD15G1365500) and RAV1 (MD13G1046100) constructs were PCR-amplified from a cDNA library derived from *Malus* crabapple leaves (cv. “Flame”) using gene-specific primers and Taq DNA polymerase (TaKaRa, Ohtsu, Japan) according to the manufacturer’s instructions. Full-length RAP2-4 and RAV1 were cloned into a modified pBI101 vector using seamless cloning at the *Nde*I and *Kpn*I sites. The PCR primers used are shown in **Table S1** (Tian et al., 2016).

*A. tumefaciens* cells were grown, collected, and resuspended to a final optical density of 1.5 at 600 nm in a solution of 10 mM MES, 10 mM MgCl_2_, and 200 mM acetosyringone and then incubated at room temperature for 3 to 4 h without shaking. The infiltration protocol and culture methods for transient expression assays in crabapple plantlets and apple fruits were adapted as previously described (Tian et al., 2016). All samples were analyzed from at least three biological replicates.

### Yeast One-Hybrid Assays

The open reading frames of RAP2-4 and RAV1 were cloned into the *Eco*RI and *Sac*I sites of pGADT7 (Clontech, Palo Alto, CA, USA) under the control of the galactokinase 4 (GAL4) promoter to yield the effector constructs. The promoter fragments of *McCHS* (MD13G1285100), *McCHI* (MD01G1117800), *McF3H* (MD02G1132200), *McDFR* (MD03G1214100), *McANS* (MD01G1153600), *McUFGT* (MD09G1141700), *McLAR1* (MD16G1048500), *McLAR2* (MD13G1046900), *McANR1* (MD10G1311100), and *McANR2* (MD05G1335600) were ligated into the pHIS2 plasmid, the sites are located upstream of the *LacZ* reporter gene (BD Biosciences, Shanghai, China). The background of the pHIS2 vectors was suppressed using 3-amino-1,2,4-triazole (3-AT). The yeast one-hybrid assay methods were as previously described (Wang N. et al., 2018). The primers used for the yeast one-hybrid assays are shown in **Table S1**.

### Accession Number

Raw sequencing data in this manuscript have been deposited in National Center for Biotechnology Information Sequence Read Archive under accession number PRJNA546094.

## Results

### Metabolic Differences Among and Transcriptome Analyses of Different Developmental Stages

**Figure 1:**
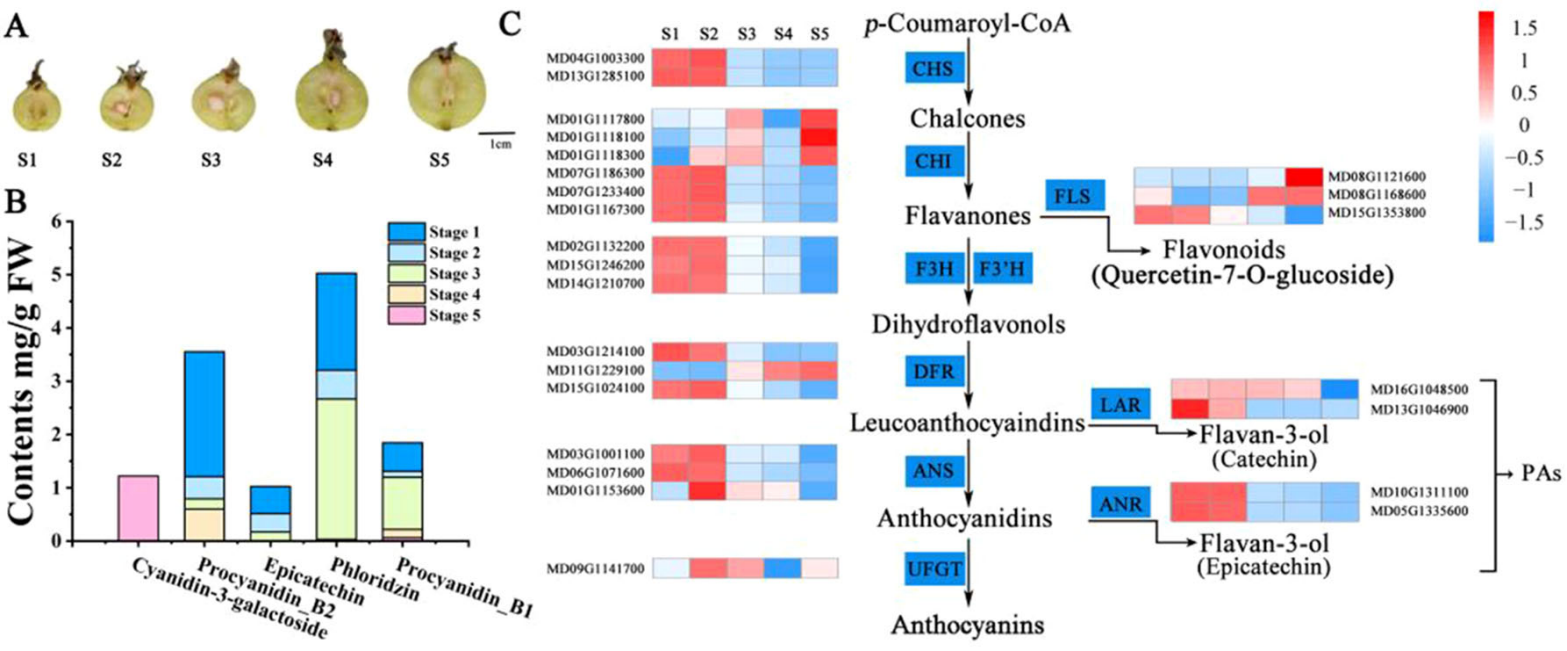
Analyses conducted for the DEGs identified by RNA-seq in the five stages of fruit development in *M*. ‘Flame’. **(A)** Fruit phenotypes at different stages (S1 to S5). **(B)** The content of the main flavonoid compounds in *M*. ‘Flame’ fruit at five developmental stages. **(C)** Expression analysis of flavonoid pathway genes at five developmental stages in fruit evaluated *via* RNA-seq with three biological replicates.

To compare the variation in flavonoid content during crabapple fruit development, we selected fruit flesh for high-performance liquid chromatography (HPLC) from three biological replicates of ‘Flame’ fruit at five different developmental stages (35, 60, 95, 120, and 150 days after full bloom) (**Figure 1A**). The main PA compounds procyanidin B1, procyanidin B2, and epicatechin were detected by HPLC, and the overall PA content (procyanidin B1, procyanidin B2, epicatechin) decreased during fruit development (**Figure 1B**). Moreover, anthocyanin accumulation was only detected at stage 5, and phloridzin accumulated in the early stages of fruit development.

We used RNA-seq analysis to profile the transcriptomes of ‘Flame’ fruit at five representative developmental stages (**Figure 1A**) (three biological replicates). The total numbers of clean reads in the RNA-seq libraries ranged from 10,664,693 to 13,963,577, and > 83% of paired reads were mapped to the apple genome (**Table 1**). Pearson correlation analysis showed that the three biological replicates had highly consistent transcriptome profiles across all developmental stages (*r* ^2^ = 0.858 to 0.986; **Figure S1**). The percentages of exonic sequences ranged from 42.95% to 56.98%, and the percentages of intronic sequences ranged from 2.19% to 5.42% (**Figure S2**).

**Table 1:** RNA sequencing data and corresponding quality control.

Sample name	Clean reads	GC content	%≧Q30	Total reads	Mapped reads
S1-1	11,910,449	3,544,170,716	47.55%	94.06%	23,820,898
S1-2	12,022,054	3,586,802,306	48.22%	93.74%	24,044,108
S1-3	12,467,852	3,716,697,858	47.98%	93.65%	24,935,704
S2-1	11,019,595	3,286,767,958	48.06%	94.76%	22,039,190
S2-2	11,266,806	3,363,171,396	47.92%	94.90%	22,533,612
S2-3	11,551,754	3,447,672,244	47.73%	95.45%	23,103,508
S3-1	11,327,223	3,369,447,850	47.48%	92.70%	22,654,446
S3-2	11,123,686	3,318,143,424	47.37%	92.39%	22,247,372
S3-3	13,008,255	3,873,955,712	47.26%	92.60%	26,016,510
S4-1	13,271,812	3,968,098,102	47.37%	95.00%	26,543,624
S4-2	13,963,577	4,172,384,900	47.44%	95.40%	27,927,154
S4-3	13,133,430	3,917,360,394	47.61%	95.46%	26,266,860
S5-1	10,664,693	3,184,130,574	47.62%	93.87%	21,329,386
S5-2	12,477,368	3,724,748,616	48.52%	93.65%	24,954,736
S5-3	11,749,880	3,507,409,072	48.42%	93.54%	23,499,760

We also observed that many flavonoid biosynthetic genes were highly expressed during development (**Figure S3**). Flavonoid pathway biosynthetic genes, including *CHS* (chalcone synthase) (MD04G1003300, MD13G1285100), *CHI* (chalcone isomerase) (MD01G1167300, MD07G1186300, MD07G1233400), flavanone 3 beta-hydroxylase (F3H, MD02G1132200, MD15G1246200), *F3H* (flavanone 3-hydrocylase) (MD02G1132200), dihydroflavonol 4-reductase/flavanone 4-reductase (DFR, MD15G1024100), flavonoid 3’-monooxygenase (F3’H, MD14G1210700, MD06G1201700), leucoanthocyanidin dioxygenase (LDOX, MD06G1071600, MD03G1001100), and flavonol synthase (FLS, MD08G1121600, MD15G1353800) showed > 11-fold differential expression (**Table S3**). Notably, the PA-related biosynthetic genes *LAR* (MD13G1046900, MD16G1048500) and *ANR* (MD05G1335600, MD10G1311100), encoding enzymes associated with PA biosynthesis, exhibited a > 20-fold decrease in expression with the development of crabapple fruit, and these results were consistent with the trends in the accumulation of the PAs (**Figure 1C**).

### Identification of DEGs Between Different Developmental Stages

To explore the molecular basis of the variations in PA content and obtain a more detailed understanding of the PA regulatory network during crabapple fruit development, the expression of each gene at the fifth developmental stage was compared to that over the consecutive developmental stages and then filtered using |log_2_(fold-change)| > 1 or < −1 and a false discovery rate (FDR) < 0.05. The most DEGs were found for S1 vs. S5 (8,710), while the S1 vs. S2 comparison had the fewest DEGs (1,645) (**Figure S3**).

**Figure 2:**
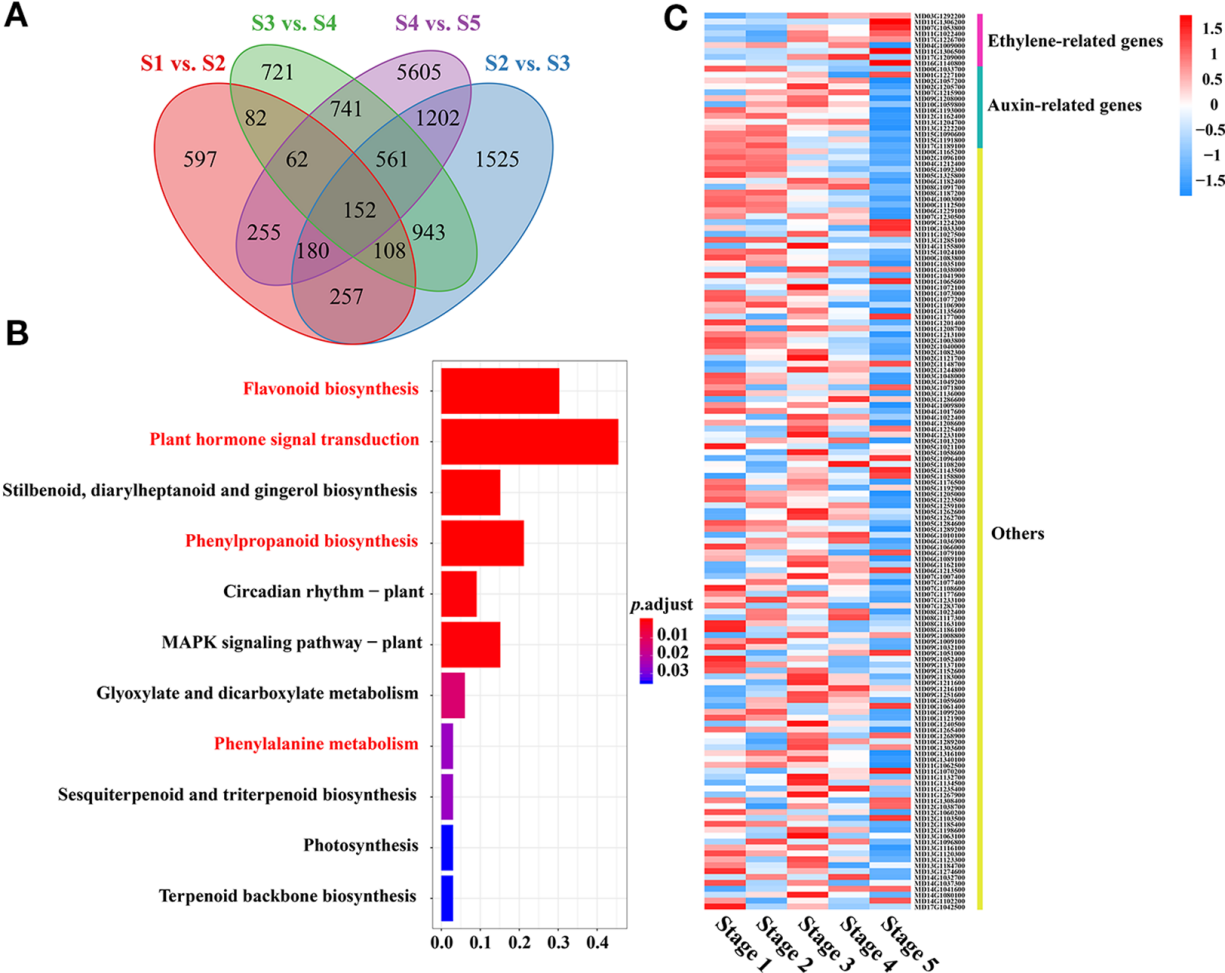
Functional analysis of DEGs between consecutive developmental stages. **(A)** Venn diagrams for the DEGs between each combination (Stage 1 vs. Stage 2, Stage 2 vs. Stage 3, Stage 3 vs. Stage 4, Stage 4 vs. Stage 5). **(B)** KEGG (Kyoto Encyclopedia of Genes and Genomes) pathway enrichment of DEGs (152) during fruit development. **(C)** Heat map comparing DEGs (152) during fruit development.

Many DEGs related to ‘phenylalanine metabolism,’ ‘phenylpropanoid biosynthesis,’ and ‘flavonoid biosynthesis,’ which are associated with PA biosynthesis, were significantly enriched during fruit development, as shown by KEGG analysis (**Figures 2A, B**). Interestingly, several DEGs related to ‘plant hormone signal transduction’ were also enriched throughout fruit development. We also identified several plant hormone signal transduction and response proteins, including auxin-responsive genes, ethylene signal transduction pathway genes and ethylene-responsive genes, among the DEGs related to ‘plant hormone signal transduction’ during fruit development (**Figure 2C**, **Table S2**). Interestingly, several auxin-responsive genes were significantly enriched throughout fruit development, which implied that auxin might be involved in PA biosynthesis during fruit flesh development in crabapple.

Notably, ethylene signal transduction pathway genes and ethylene-responsive genes, including ethylene-responsive transcription factors and the ethylene receptor, were upregulated in a stage-specific manner during the later stages of fruit flesh development. In addition, many DEGs related to ethylene biosynthesis and signal transduction, such as ERS (ethylene response sensor) (MD03G1292200), EIN3 (ETHYLENE INSENSITIVE 3) (MD07G1053800), and ERFs (ethylene response factors) (MD04G1009000, MD11G1306500, MD17G1209000, MD16G1140800), were enriched during fruit development, suggesting that ethylene may play an important role in regulating PA biosynthesis in the late stages of fruit flesh development.

### Identification of WGCNA (Weighted Correlation Network Analysis) Modules and Hub Genes Associated With PA Biosynthesis

**Figure 3:**
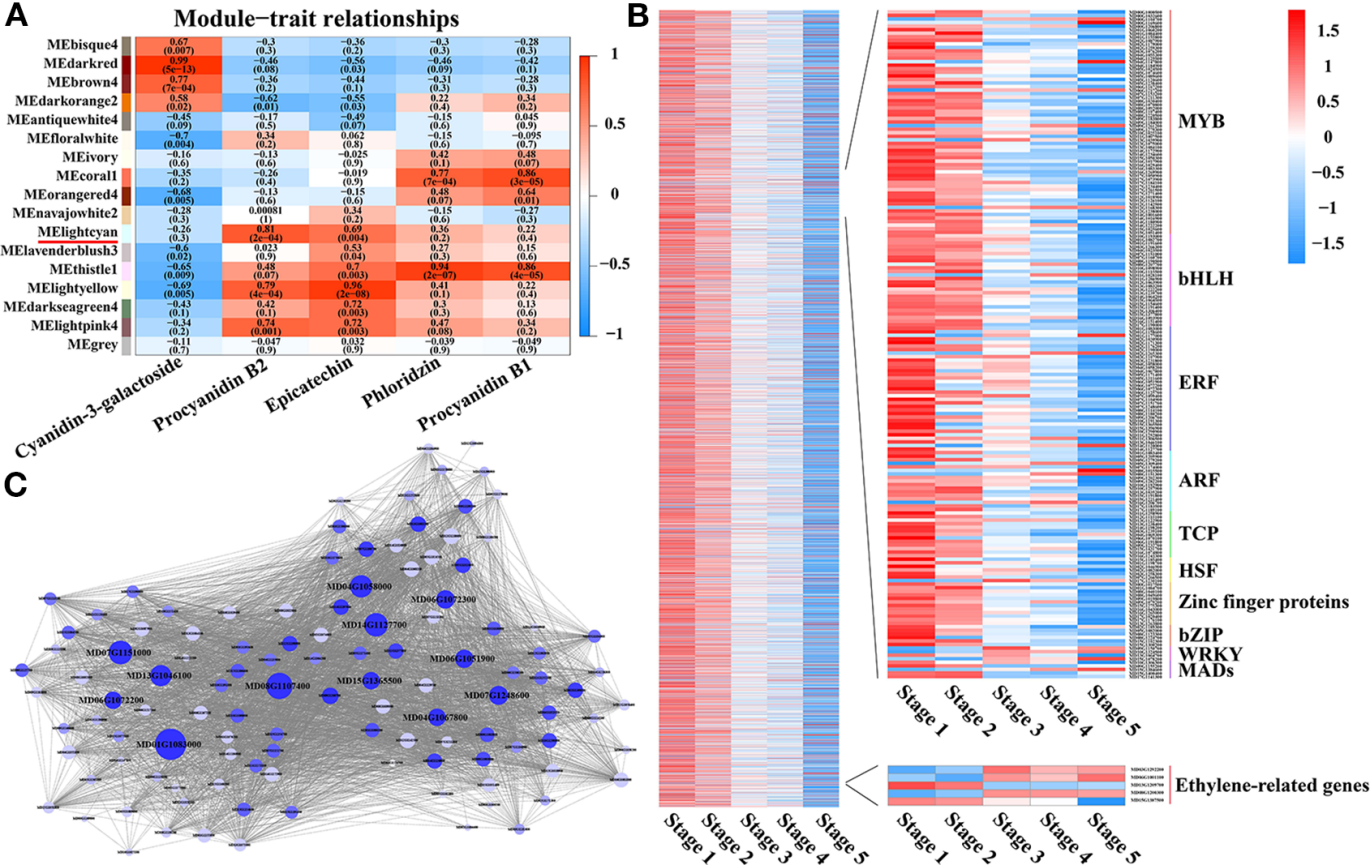
Identification of WGCNA modules and hub genes associated with proanthocyanidin biosynthesis. **(A)** Module-PA weight correlations and corresponding *P*-values (in parentheses). A high degree of correlation between a specific module and the procyanidin B2 is indicated by red underlining of the module name. **(B)** Transcriptional heat map of genes in the MElightcyan module, the module with the highest correlation with procyanidin B2. **(C)** Analysis of TF correlation networks in the MElightcyan module. Candidate hub genes are shown in bigger font, and the size of the graph is positively correlated with the PA correlation. Different letters above the bars indicate significantly different values (*P* < 0.05) calculated using one-way analysis of variance (ANOVA) followed by Tukey’s multiple range test.

To further identify the specific transcription factors involved in regulating PA biosynthesis during crabapple fruit development, a total of 9,471 DEGs were used in a WGCNA analysis, resulting in 17 distinct modules (**Figure 3A**). The MElightcyan module was the highest correlative module with procyanidin B2, and this module included 4,297 genes and had the highest correlation with PA accumulation (0.81) across all developmental stages (**Figure 3B**). In the MElightcyan module, 137 and 296 genes were related to ‘signal transduction mechanisms’ and ‘transcription,’ respectively (**Figure 3B**, **Tables S3** and **S4**). These analyses further showed that as the fruit developed, ethylene signal transduction pathway genes gradually increased, while ethylene-responsive genes gradually decreased. In addition, genes associated with ‘transcription’ were highly enriched for the MYB, bHLH, ERF, and ARF families. These data indicate that ethylene signal transduction pathway genes or ethylene-responsive genes play an important role in the regulatory network of PA biosynthesis and that MYBs, bHLHs, ERFs, and ARFs may be involved in the metabolism of PAs.

To further identify the specific TFs that participate in regulating PA biosynthesis during crabapple fruit development, 296 genes encoding transcription factors, including members of the MYB, bHLH, ERF, TCP, bZIP, WRKY, and WD40 families, were further analyzed *via* a correlation network (**Figure 3C**). The top 150 genes that showed the most connections in the network based on their high K_ME_ (eigengene connectivity) values were defined as hub genes, and the enrichment for MYB, bHLH, and ERF transcription factors were detected.

**Figure 4:**
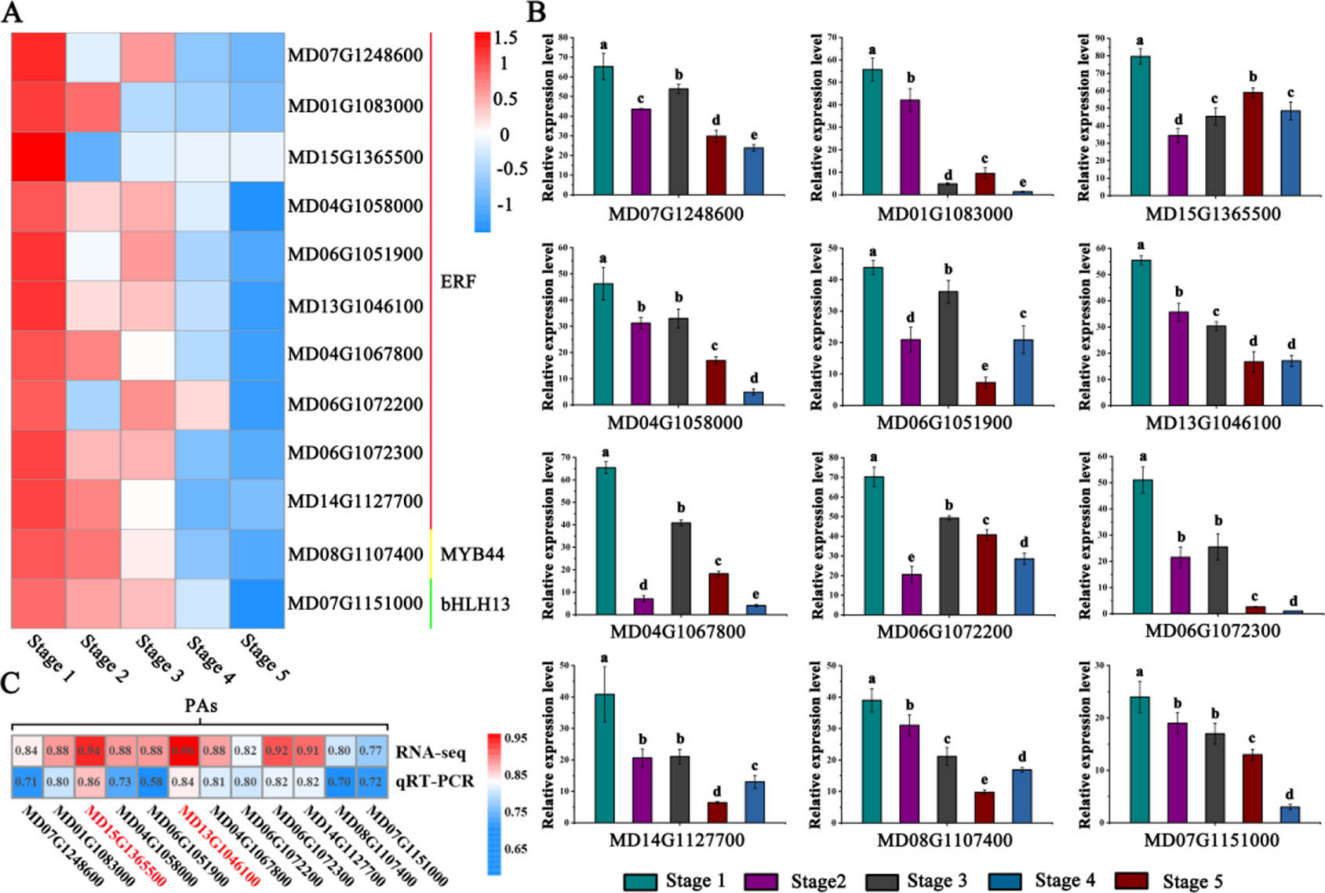
Identification and analysis of PA-biosynthesis-associated transcription factor genes. **(A)** Heat maps describing the expression profiles of candidate genes related to PA biosynthesis. ERF represents ethylene-responsive transcription factors, MYB represents the R2R3-MYB transcription factor, bHLH represents the helix-loop-helix DNA-binding domain. **(B)** Validation of RNA-seq expression profiles *via* qRT-PCR. **(C)** Correlation analysis between PA accumulation and the expression of related candidate PA regulators *via* RNA-seq and qRT-PCR data. Different letters above the bars indicate significantly different values (*P* < 0.05) calculated using one-way analysis of variance (ANOVA) followed by Tukey’s multiple range test.

Through correlation networks and gene expression trends, we identified 12 transcription factors from the MYB, bHLH and ERF families as hub genes (**Figure 3C**). These genes included ethylene-response factors (ERF105, MD07G1248600; ERF023, MD01G1083000; RAP2-4, MD15G1365500; ERF1A, MD04G1058000; ERF5, MD06G1051900; RAV1, MD13G1046100; DREB1A, MD06G1072300; DREB1D, MD04G1067800; ERF1E, MD06G1072200; and ERF061, MD14G1127700), MYB44 (MD08G1107400) and bHLH13 (MD07G1151000) (**Figure 4A**).

To visualize the reliability of the RNA-seq data, the expression level of hub genes were detected by qRT-PCR analysis (**Figure 4B**). The results showed that the expression levels of these genes gradually decreased during fruit development. To better validate the reliability of the selected hub genes, we generated a heat map showing the correlation data. The RNA-seq and qRT-PCR data showed strong correlations (> 0.70) between PAs and the expression of related candidate PA regulators (**Figure 4C**).

### Functional Assay of Transcription Factors Associated With PA Biosynthesis in Crabapple Plantlets and Apple Fruits

The ERF family gene *RAP2-4* (RELATED TO APETALA 2-4) participates in regulating plant development and stress resistance *via* light perception and ethylene signaling (Lin et al., 2008). By contrast, *RAV1* (related to ABI3/VP1) is known to be a suppressor involved in flower development, growth, and stress responses (Hu et al., 2004). Here, the expression of *RAP2-4* and *RAV1* showed the greatest correlations with PAs accumulation among the 12 identified TFs during fruit development (**Figures 4A, C**).

To assess the role of *RAP2-4* and *RAV1* in PA biosynthesis, 1221 bp *PAP2* and 1098 bp *RAV1* cDNA sequences from ‘Royalty’ were cloned from fruit flesh, and they were predicted to encode 406 and 365 amino acids, respectively.

**Figure 5:**
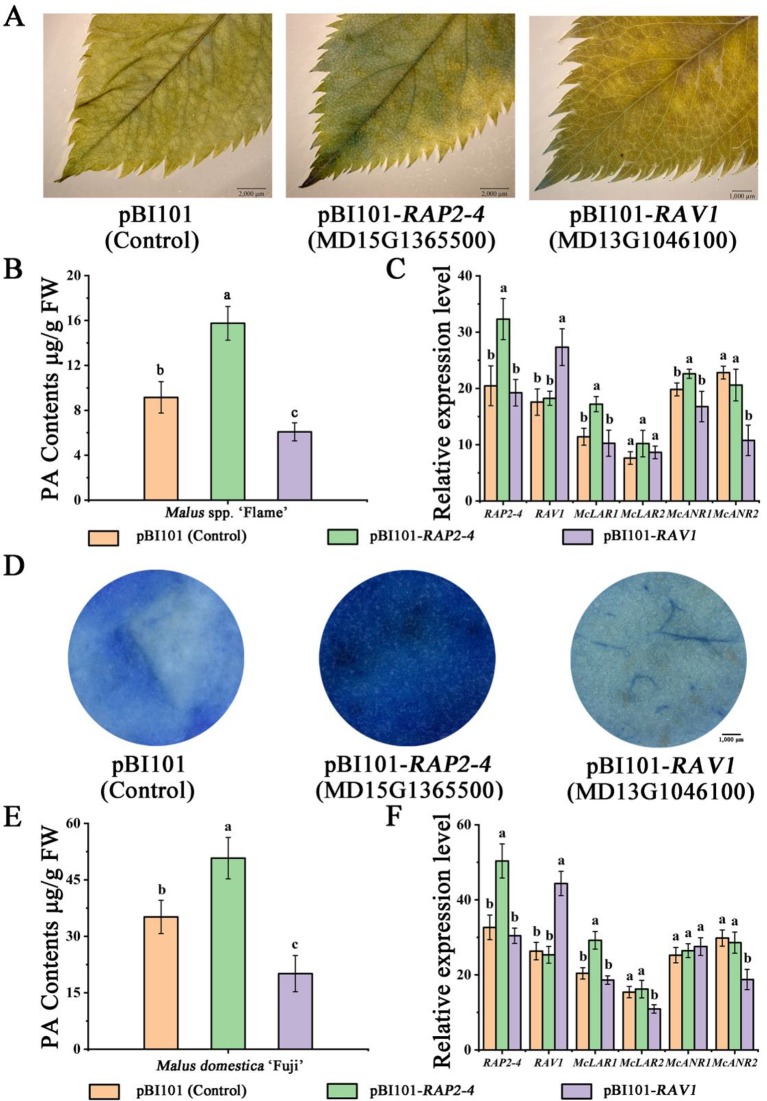
Overexpression of RAP2-4 (MD15G1365500) and RAV1 (MD13G1046100) in *Malus* crabapple leaves and *Malus* domestica ‘Fuji’ fruit peels. **(A)** 4-Dimethylaminocinnamaldehyde (DMACA) staining in pRI101-, RAP2-4 (MD15G1365500)-, and RAV1 (MD13G1046100)- overexpressing crabapple leaves. **(B)** The PA contents of transiently overexpressing leaves. **(C)** Expression analysis of RAP2-4, RAV1, and PA-related biosynthetic genes in transiently overexpressing leaves. **(D)** DMACA staining in pRI101-, RAP2-4 (MD15G1365500)-, and RAV1 (MD13G1046100)-overexpressing apple peels. **(E)** The PA contents of transiently overexpressing apple peels. **(F)** Expression analysis of *RAP2-4*, *RAV1*, and PA-related biosynthetic genes in transiently overexpressing apple peels. All results are derived from three biological replicates. Different letters above the bars indicate significantly different values (*P* < 0.05) calculated using one-way analysis of variance (ANOVA) followed by Tukey’s multiple range test.

Subsequently, the vector constructs *35S*::*RAP2-4* and *35S*::*RAV1* (pBI101 for overexpression) were overexpressed in ‘Flame’ tissue culture plants, resulting in stronger DMACA staining and higher PA content in *35S*::*RAP2-4* expressed leaves than in the control (**Figure 5A**). We also observed weaker DMACA staining and lower PA content in *RAV1*-overexpressing leaves than in control leaves (**Figure 5A**). Gene expression analysis by qRT-PCR further indicated that the expression of the PA-related genes *McLAR1* and *McANR1* increased compared to those in the control in *RAP2-4*-overexpressing leaves, while *McANR2* expression was lower in *RAV1*-overexpressing leaves than in the control (**Figures 5B, C**).

We also overexpressed the *RAP2-4* and *RAV1* genes in ‘Red Fuji’ fruits to further reveal the role of these two TFs. *Agrobacterium tumefaciens* cultures containing *35S*::*RAP2-4* or *35S*::*RAV1* were individually injected into apple fruits. The fruits infiltrated with *35S*::*RAP2-4* rapidly accumulated PAs, resulting in deep blue staining in the peels. In contrast, apple fruits infiltrated with the *35S*::*RAV1* showed weaker DMACA staining. (**Figure 5D**). By using qRT-PCR, the expression of *McLAR1* strongly increased compared to control peels in *RAP2-4*-overexpressing fruit peels, we also observed a significant decrease in the expression of *McLAR2* and *McANR2* when *RAV1* was overexpressed. (**Figures 5E, F**). These results suggest that RAP2-4 may act as an activator in PA biosynthesis, while RAV1 acts as a suppressor.

### RAP2-4 and RAV1 Proteins Bind to the Promoters of PA Biosynthetic Genes

**Figure 6:**
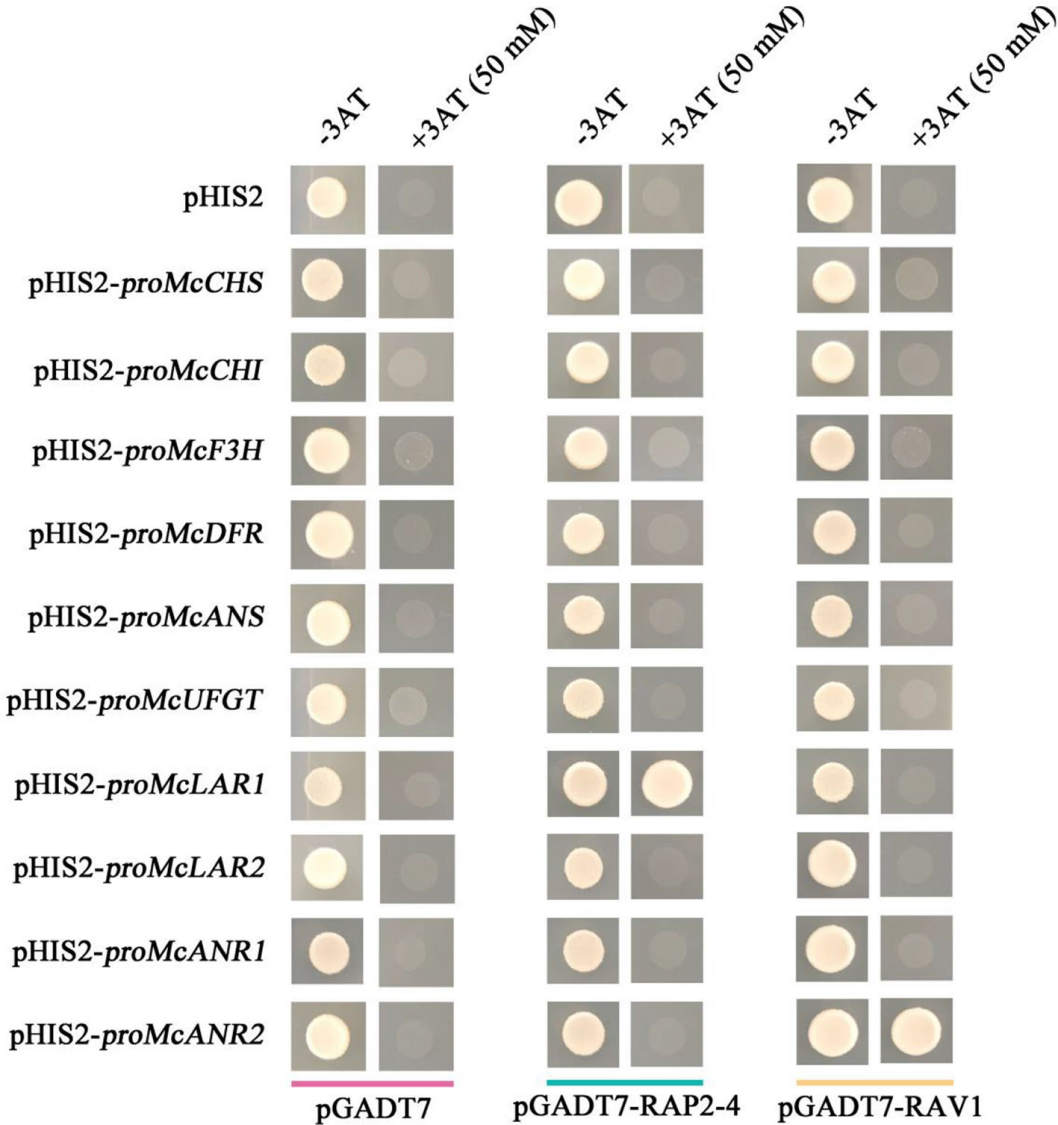
*Cis*-element binding ability of RAP2-4 and RAV1 with anthocyanin and PA biosynthetic genes. Interaction of RAP2-4 and RAV1 proteins with the promoters of flavonoid biosynthetic genes as revealed by yeast one-hybrid assays. The concentration of 3AT is 50 mM for RAP2-4 and RAV1. Yeast transformed with pGADT7/pHIS2, pGADT7-RAP2-4/pHIS2, pGADT7-RAV1/pHIS2, or pGADT7-/pHIS2-flavonoid biosynthetic gene promoters were used as controls.

To verify the speculation that PA biosynthetic genes might be regulated by RAP2-4 and RAV1 in crabapple, a yeast one-hybrid assay was employed to test their ability to bind the promoters of *McCHS*, *McCHI*, *McF3H*, *McDFR*, *McANS*, *McUFGT*, *McLAR1*, *McLAR2, McANR1*, and *McANR2*. The results showed that RAP2-4 bound the promoter of *McLAR1*, and RAV1 bound the promoter of *McANR2*. From these results, we deduced that the PA biosynthetic genes *McLAR1* and *McANR2* might be candidate target genes of *RAP2-4* and *RAV1*, respectively (**Figure 6**).

## Discussion

PAs accumulate in several plant tissues, especially in fruits, and play many important roles in physiological and developmental processes; PAs are the main products of the flavonoid pathway and have significant health benefits to humans (Bondonno et al., 2019). Crabapple fruits produce abundant PA compounds, making them a valuable model for studying the molecular mechanisms of PA biosynthesis. In our study, we found *via* HPLC analysis that the accumulation of PAs was enriched in young fruits during fruit development. Similar trends in expression have previously been reported for banana fruit (*Musa*); the expression levels of *MaANR* and *MaLAR* were associated with the accumulation of PAs in young fruits (Pandey et al., 2016). Thus, we used RNA-seq analysis to focus on the molecular mechanisms underlying PA biosynthesis during fruit development.

Abscisic acid, ethylene, and jasmonic acid have been reported to be involved in anthocyanin biosynthesis and to promote fruit ripening, while auxin and gibberellin inhibit anthocyanin biosynthesis and delay fruit maturation (Jaakola, 2013; Murcia et al., 2016; Olivares et al., 2017). Moreover, previous research has shown that the ABA signaling network promotes flavonol biosynthesis (especially of quercetin derivatives) in early land plants (Brunetti et al., 2019). These results showed that phytohormones play important roles in the development and ripening of fruit and are also involved in flavonoid biosynthesis (Coelho et al., 2019). In our research, many DEGs related to ‘plant hormone signal transduction’ were enriched throughout fruit development, suggesting a correlation between plant hormones and PA biosynthesis in *Malus* crabapple fruit (**Figure 2**). Further analyses of DEGs related to ‘plant hormone signal transduction’ identified auxin and ethylene as playing important roles during the development of fruit flesh, and they may be involved in the regulation of PA biosynthesis. Moreover, many DEGs encoding genes involved in ethylene biosynthesis and ethylene signal transduction, as well as ERF transcription factors, were significantly enriched during fruit development, suggesting a correlation between ethylene and PA biosynthesis in *Malus* crabapple fruit.

Further analyses with the MElightcyan module, the module with the highest correlation with procyanidin B2, showed that ethylene signal transduction pathway and ethylene response genes play an important role in the regulatory network of PA biosynthesis, and MYBs, bHLHs, ERFs, and ARFs may be involved in PA metabolism (**Figure 3**). This information suggests a new path for exploring the PA regulatory network. Several studies have reported that the regulation of genes involved in PA biosynthesis is in part mediated by many transcription factors. MYB and bHLH transcription factors have been the most comprehensively researched, including in Arabidopsis (Nesi et al., 2001), strawberry (Schaart et al., 2013), grape (Deluc et al., 2006), persimmon (Akagi et al., 2009) and apple (Wang et al., 2017). In addition, MdARF13 interacts with MdMYB10 to promote anthocyanin biosynthesis by directly binding the promoter of *MdDFR*. By contrast, MdERF1B is responsible for regulating anthocyanin and PA accumulation, mainly by acting on MdMYB9 and MdMYB11 in apple (Zhang et al., 2018; Wang Y. C. et al., 2018). In our study, the genes encoding MYB, bHLH, and ERF transcription factors exhibited significantly higher expression and showed the same trends as PA accumulation in the 9,471 DEGs identified *via* WGCNA and transcription factor correlation network analysis (**Figure 3**). We hypothesized that the MYB, bHLH, and ERF families play pivotal roles in the regulation of PA biosynthesis in *Malus* crabapple and selected 12 candidate hub genes from these families for subsequent verification (**Figure 4**).

Ethylene, the major ripening hormone in climacteric fruit, is also involved in regulating flavonoid biosynthesis (Onik et al., 2018; Li et al., 2016). In grape berries, exogenous ethylene promotes anthocyanin biosynthesis by stimulating the expression of *CHS*, *F3H*, *LDOX*, and *UFGT* (El-Kereamy et al., 2003). In plum, ethylene-treated fruits show significantly improved flesh reddening *via* an increase in *PAL* expression (Manganaris et al., 2008). In apples, exogenous ethylene treatment during fruit ripening increased the anthocyanin content and the enzymatic activity of anthocyanin biosynthetic genes (Faragher and Brohier, 1984). However, it is unclear whether ethylene promotes PA accumulation during fruit ripening. On the other hand, ERF TFs have been identified as regulators of flavonoid biosynthesis in plants. In Arabidopsis, AtERF4 and AtERF8 promote anthocyanin accumulation by activating the expression of anthocyanin biosynthetic genes under light treatment (Koyama and Sato, 2018). In pear (*Pyrus bretschneideri*), PyERF3 enhances anthocyanin biosynthesis by interacting with both PyMYB114 and PybHLH3, and Pp4ERF24 and PpERF96 interact with MYB114 and participate in blue-light-mediated anthocyanin biosynthesis in pear fruits (Yao et al., 2017; Ni et al., 2019). In apple, MdERF3 promotes anthocyanin biosynthesis by interacting with MdMYB1 (An et al., 2018b), while MdERF1B has been reported to interact with MdMYB9, MdMYB1, and MdMYB11 to regulate anthocyanin and proanthocyanidin biosynthesis (Zhang et al., 2018).

In our study, by using WGCNA, we noticed several ERF TFs in the MElightcyan module. Considering the important roles of ethylene during fruit development, we selected two ERF transcription factors (*RAP2-4*, MD15G1365500, and *RAV1*, MD13G1046100) that had the greatest difference in expression for further analysis. Previous studies have shown that *RAP2-4* plays critical roles in waterlogging tolerance, cold and heat stress, salt stress, and drought stress (Figueroayañez et al., 2016; Phukan et al., 2018). In addition, RAV1 acts as a negative regulator of growth in many plant species, and its transcription is downregulated by BR and ABA (Hu et al., 2004). Expression analysis was employed to detect the functional role of these two ERF TFs in crabapple fruits. The results showed that the transcription levels of RAP2-4 and RAV1 were positively correlated with the PA contents of crabapple fruits. Y1H suggested that these two ERF TFs participate in regulating PA accumulation by binding to the promoters of PA biosynthetic genes. These results were further confirmed by transient overexpression analysis. Thus, we deduced that RAP2-4 and RAV1 may be candidate PA regulators and play pivotal roles in regulating PA biosynthesis during crabapple fruit development. Furthermore, we found that RAP2-4 acts as a positive regulator and that RAV1 acts as a negative regulator in PA accumulation. We speculated that these two TFs may participate in PA biosynthesis *via* competitive interaction with MYB or bHLH TFs, and this hypothesis will be addressed in future studies.

Overall, RNA-seq analyses and the functional studies of these two ERF transcription factors provide insights into fruit development. Notably, we found that ethylene plays a critical role in this pathway and that ERF transcription factors regulate PA biosynthesis.

## Data Availability Statement

The datasets generated for this study can be found in the NCBI SRA database: PRJNA546083.

## Author Contributions

Conceived of and designed the experiments: JT, YY. Performed the experiments: HL, MH, LY, SW. Analyzed the data: MH, JT, YY. Contributed reagents/materials/analytic tools: JZ, JT, YY. Wrote the paper: HL, JT, YY.

## Conflict of Interest

The authors declare that the research was conducted in the absence of any commercial or financial relationships that could be construed as a potential conflict of interest.
