# Supplementary material for: Transcriptome Analysis Identifies Two Ethylene Response Factors That Regulate Proanthocyanidin Biosynthesis During Malus Crabapple Fruit Development
Source: Front Plant Sci. 2020 Feb 26;11:76. doi: 10.3389/fpls.2020.00076 (PMC7054237; doi:10.3389/fpls.2020.00076)
Supplement: Supplementary file 1 [file DataSheet_1.doc]

**Supplementary Materials**

**
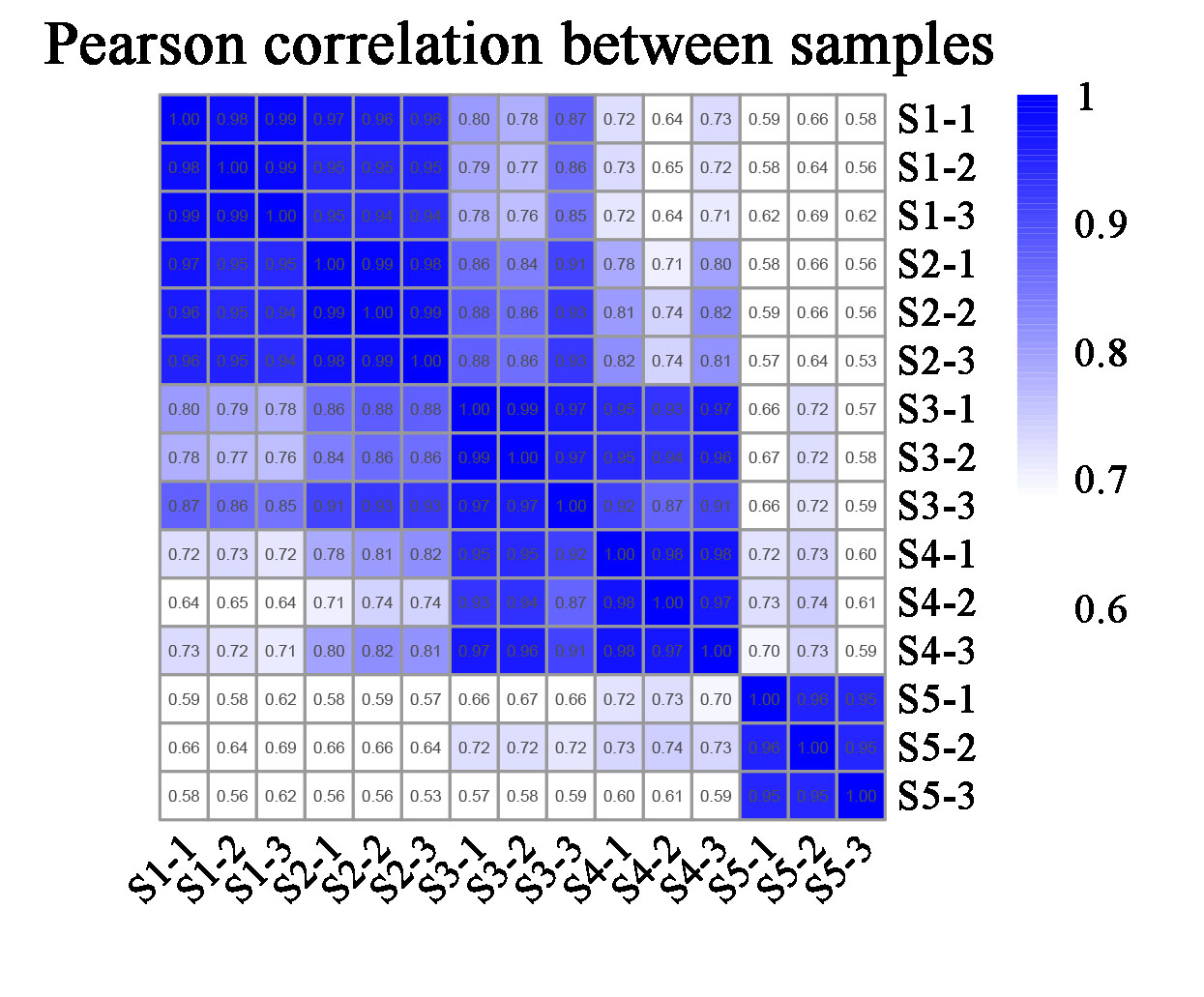
**

**Fig. S1. Heat map showing the correlations between biological replicates.** The PCC (Pearson correlation coefficient) values are quantitative indicators of relative expression levels of all genes in each sample.





**Fig. S2. The distribution of cleaned RNA-seq reads mapped to the apple reference genome.** ‘Exon’, ‘intron’ and ‘intergenic’ refer to the percentages of cleaned reads mapped to an exon intron or intergenic region, respectively, in the reference genome.

**
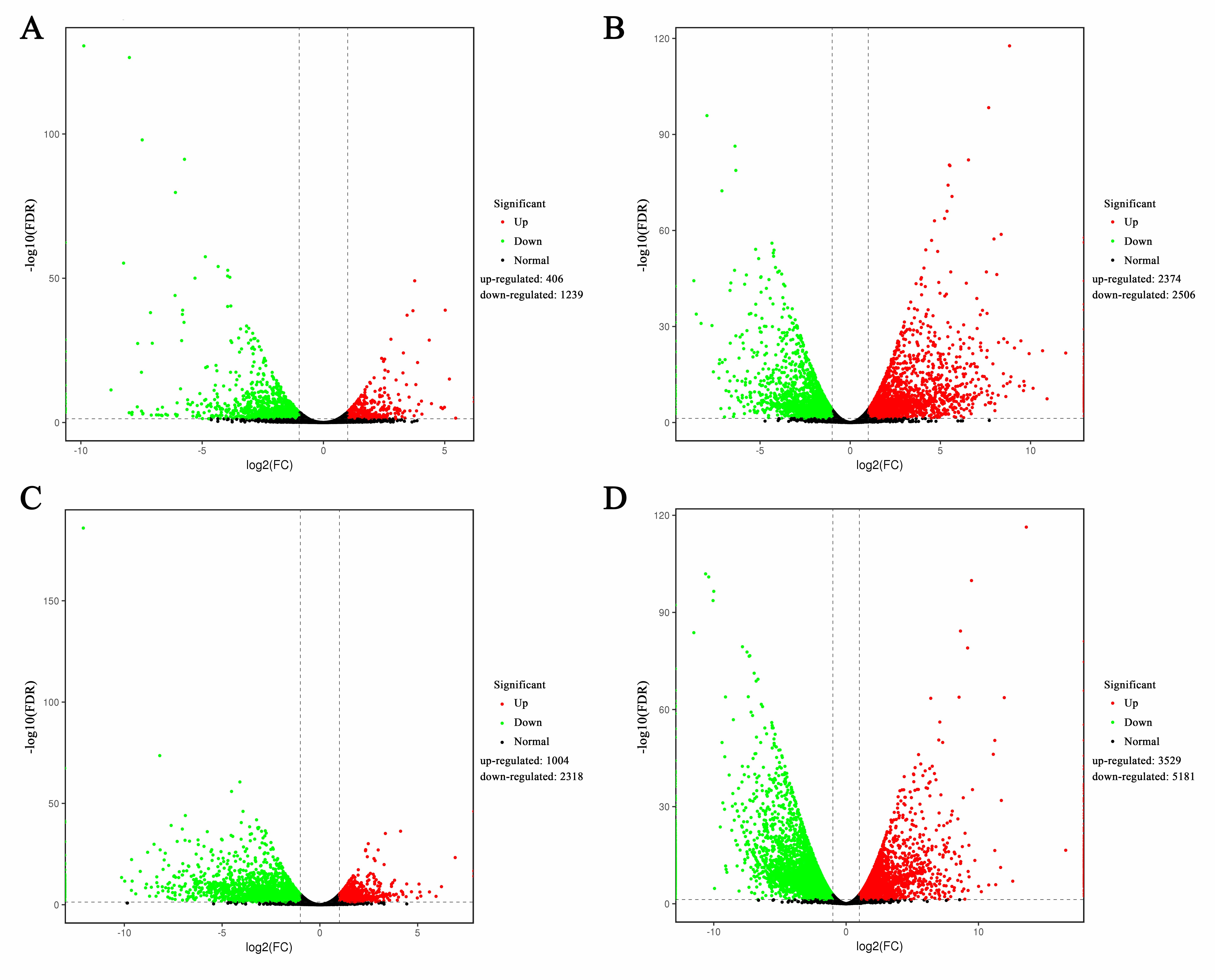
**

**Fig. S3. Volcano plot visualizing the DEGs between two contiguous developmental stage. (A-D)** Volcano plot of DEGs in Stage 1 vs. Stage 2 **(A)**, Stage 2 vs. Stage 3 **(B)**, Stage 3 vs. Stage 4 **(C)**, Stage 4 vs. Stage 5 **(D)**, respectively.

| **Table S1.** DNA primers used in this study. | | |
| --- | --- | --- |
| **Primer name** | **Primer Sequence(5'-3')** | **Used for** |
| q-MD06G1072200-F | AATGACCTCCTCGTCGGAGA | RT-qPCR analysis |
| q-MD06G1072200-R | CACTCTCAACTCACGGCTCA | RT-qPCR analysis |
| q-MD13G1046100-F | AGGTTGGTCCGGTTCCGATGG | RT-qPCR analysis |
| q-MD13G1046100-R | ACCTCCGCCGATAGCAGCAG | RT-qPCR analysis |
| q-MD07G1248600-F | TCTGGAGCTGATTAGGCGACACC | RT-qPCR analysis |
| q-MD07G1248600-R | GTGGTGGTGGAGGAGGAGGAAG | RT-qPCR analysis |
| q-MD06G1072300-F | GGCGATGATGAGAAGGAGAGCAAG | RT-qPCR analysis |
| q-MD06G1072300-R | CTTCCGCCATACTATCCAGCAACC | RT-qPCR analysis |
| q-MD01G1083000-F | GACGACGACGACGATCATCACG | RT-qPCR analysis |
| q-MD01G1083000-R | ACGCAGTCATATTCCAGCCACATG | RT-qPCR analysis |
| q-MD15G1365500-F | CTTCCTCCTCCTCCTACGACTTCC | RT-qPCR analysis |
| q-MD15G1365500-R | GACGGCAGTTGCTGGTGGTG | RT-qPCR analysis |
| q-MD04G1058000-F | GTCATCACCAGAACCGTCGTCTTC | RT-qPCR analysis |
| q-MD04G1058000-R | GTAACCGACACCGCCACTTGG | RT-qPCR analysis |
| q-MD04G1067800-F | GTCCAGCACGCCGAAGAAGC | RT-qPCR analysis |
| q-MD04G1067800-R | TCGTTGTTCCTCCTCCTCACTCC | RT-qPCR analysis |
| q-MD06G1051900-F | GAAGATGCGAAGGCGGTGGTG | RT-qPCR analysis |
| q-MD06G1051900-R | ACGGCTGGATGAGGAGATAACGG | RT-qPCR analysis |
| q-MD08G1107400-F | GGCGGCGGCAATGTGAGTAG | RT-qPCR analysis |
| q-MD08G1107400-R | CCGACGGCACACTCTCATTATTCC | RT-qPCR analysis |
| q-MD15G1344900-F | ACCGCTCGCCGAGGAAGAAG | RT-qPCR analysis |
| q-MD15G1344900-R | TGTTGTTCTTGCTGCTGCTGAGG | RT-qPCR analysis |
| q-MD07G1151000-F | ACCAGGCACCTGATGTTGATGTTC | RT-qPCR analysis |
| q-MD07G1151000-R | CTGCTCCGATCCTTGAGACTTGAC | RT-qPCR analysis |
| q-MD01G1082700-F | ACCAGGCACCTGATGTTGATGTTC | RT-qPCR analysis |
| q-MD01G1082700-R | CTGCTCCGATCCTTGAGACTTGAC | RT-qPCR analysis |
| q-MD14G1127700-F | TTCACAGACGCCATTGACATCCG | RT-qPCR analysis |
| q-MD14G1127700-R | CCGCTGGTGGTGGTTGTTACG | RT-qPCR analysis |
| pBI101-RAV1-F | AGCAAGTTCTTCACTGTTGATACATATGGACGGAATAAGCAGCACAGAAG | Overexpression assays |
| pBI101-RAV1-R | CCTTGCTCACCATGGATCCGGTACCCAAAGCTCCAATGATCCTTGGC | Overexpression assays |
| pBI101-RAP2-4-F | AGCAAGTTCTTCACTGTTGATACATATGGCAACTACAATGGATTTCTACA | Overexpression assays |
| pBI101-RAP2-4-R | CCTTGCTCACCATGGATCCGGTACCCTGAAGATCGAAGCCCAATCAA | Overexpression assays |
| pGADT7-RAV1-F | ATACGACGTACCAGATTACGCTCATATGGACGGAATAAGCAGCACAGAAG | Yeast one-hybrid assays |
| pGADT7-RAV1-R | CGTATCGATGCCCACCCGGGTGGAACAAAGCTCCAATGATCCTTGGC | Yeast one-hybrid assays |
| pGADT7-RAP2-4-F | ATACGACGTACCAGATTACGCTCATATGGCAACTACAATGGATTTCTACA | Yeast one-hybrid assays |
| pGADT7-RAP2-4-R | CGTATCGATGCCCACCCGGGTGGAACTGAAGATCGAAGCCCAATCAA | Yeast one-hybrid assays |
| pHIS2-*ProMcCHS*-F | TTGTAATACGACTCACTATAGGGCG ATAGAAACCAGCTTGACACAGCTGA | Yeast one-hybrid assays |
| pHIS2-*ProMcCHS*-R | GGATCGATTCGCGAACGCGTGAGCT TTTATCGGGTCGTCAAGAAAGATCA | Yeast one-hybrid assays |
| pHIS2-*ProMcCHI*-F | TTGTAATACGACTCACTATAGGGCG GTGGTGGAGGTCACGGTGGGGTGTG | Yeast one-hybrid assays |
| pHIS2-*ProMcCHI*-R | GGATCGATTCGCGAACGCGTGAGCT TGACAATTTTGTTGTTATTGTGTAG | Yeast one-hybrid assays |
| pHIS2-*ProMcF3H*-F | TTGTAATACGACTCACTATAGGGCG AATGTATGGTCTGCGTTTCTCCATT | Yeast one-hybrid assays |
| pHIS2-*ProMcF3H*-R | GGATCGATTCGCGAACGCGTGAGCT TTTGCTTAGACTTCTCCCCAGCCAC | Yeast one-hybrid assays |
| pHIS2-*ProMcDFR*-F | TTGTAATACGACTCACTATAGGGCG AATCAGAATGCCACGAACCACTCAG | Yeast one-hybrid assays |
| pHIS2-*ProMcDFR*-R | GGATCGATTCGCGAACGCGTGAGCT TCTTGTGTGTATGTGCTTACCGAAG | Yeast one-hybrid assays |
| pHIS2-*ProMcANS*-F | TTGTAATACGACTCACTATAGGGCG TCAACCTCCGGTCGCATGATGAAAG | Yeast one-hybrid assays |
| pHIS2-*ProMcANS*-R | GGATCGATTCGCGAACGCGTGAGCT GAAGGGAAAAAACTGCTGCTAGCTA | Yeast one-hybrid assays |
| pHIS2-*ProMcUFGT*-F | TTGTAATACGACTCACTATAGGGCG TCTAAGCTTCTAACTCATCGACTTT | Yeast one-hybrid assays |
| pHIS2-*ProMcUFGT*-R | GGATCGATTCGCGAACGCGTGAGCT ACAGCTTACAAGGCTAATTAGAAAA | Yeast one-hybrid assays |
| pHIS2-*ProMcLAR1*-F | TTGTAATACGACTCACTATAGGGCG CCTTTTTTCAACCTTATCGGGTGGA | Yeast one-hybrid assays |
| pHIS2-*ProMcLAR1*-R | GGATCGATTCGCGAACGCGTGAGCT GGCTGCTGCTGCTCTTCTTTCCTGC | Yeast one-hybrid assays |
| pHIS2-*ProMcLAR2*-F | TTGTAATACGACTCACTATAGGGCG CTAGGGATGTGTAGTAACTGGCCAA | Yeast one-hybrid assays |
| pHIS2-*ProMcLAR2*-R | GGATCGATTCGCGAACGCGTGAGCT AAGGCAAAGGGAAGGCCCTTTGATG | Yeast one-hybrid assays |
| pHIS2-*ProMcANR1*-F | TTGTAATACGACTCACTATAGGGCG ATTCAATTCTGAGTACTGTAACCTA | Yeast one-hybrid assays |
| pHIS2-*ProMcANR1*-R | GGATCGATTCGCGAACGCGTGAGCT GCTTCCTCCTCCTCTGTCAGATTTT | Yeast one-hybrid assays |
| pHIS2-*ProMcANR2*-F | TTGTAATACGACTCACTATAGGGCG ACATCAGAGTGGTTCCTTAAACAAT | Yeast one-hybrid assays |
| pHIS2-*ProMcANR2*-R | GGATCGATTCGCGAACGCGTGAGCT TGGCTTCCACTTCCTCTATCAAAGT | Yeast one-hybrid assays |

| **Table S2. List of DGEs (152) during the development of fruit.**   | **Gene_id** | **NR_annotation** | | --- | --- | | MD00G1033700 | sp|O65695|SAU50_ARATH Auxin-responsive protein SAUR50 OS=*Arabidopsis thaliana* GN=SAUR50 PE=1 SV=1 | | MD00G1083800 | sp|O23006|LYM2_ARATH LysM domain-containing GPI-anchored protein 2 OS=*Arabidopsis thaliana* GN=LYM2 PE=1 SV=1 | | MD00G1112500 | sp|Q43873|PER73_ARATH Peroxidase 73 OS=*Arabidopsis thaliana* GN=PER73 PE=1 SV=1 | | MD00G1165200 | sp|Q9SKE2|JAR1_ARATH Jasmonic acid-amido synthetase JAR1 OS=*Arabidopsis thaliana* GN=JAR1 PE=1 SV=2 | | MD01G1035100 | sp|Q9FJN7|GCL1_ARATH LanC-like protein GCL1 OS=*Arabidopsis thaliana* GN=GCL1 PE=2 SV=1 | | MD01G1038000 | sp|Q9SJY5|PUMP5_ARATH Mitochondrial uncoupling protein 5 OS=*Arabidopsis thaliana* GN=PUMP5 PE=2 SV=1 | | MD01G1041900 | sp|Q96289|ZAT10_ARATH Zinc finger protein ZAT10 OS=*Arabidopsis thaliana* GN=ZAT10 PE=2 SV=1 | | MD01G1065600 | sp|Q1KLZ1|CAS2_MALDO L-3-cyanoalanine synthase 2, mitochondrial OS=*Malus domestica* GN=CAS2 PE=1 SV=1 | | MD01G1072100 | sp|Q6DST1|Y1465_ARATH Late embryogenesis abundant protein At1g64065 OS=*Arabidopsis thaliana* GN=At1g64065 PE=2 SV=1 | | MD01G1073000 | sp|Q3EBF7|SLD2_ARATH Delta(8)-fatty-acid desaturase 2 OS=*Arabidopsis thaliana* GN=SLD2 PE=1 SV=1 | | MD01G1077200 | sp|Q9AR73|HQGT_RAUSE Hydroquinone glucosyltransferase OS=*Rauvolfia serpentina* GN=AS PE=1 SV=1 | | MD01G1106900 | sp|Q9SMK9|PAL2_CICAR Phenylalanine ammonia-lyase 2 OS=*Cicer arietinum* GN=PAL2 PE=2 SV=1 | | MD01G1135600 | sp|O48818|EXPA4_ARATH Expansin-A4 OS=*Arabidopsis thaliana* GN=EXPA4 PE=1 SV=1 | | MD01G1162100 | sp|A7NY33|PER4_VITVI Peroxidase 4 OS=*Vitis vinifera* GN=GSVIVT00023967001 PE=1 SV=1 | | MD01G1162400 | sp|A7NY33|PER4_VITVI Peroxidase 4 OS=*Vitis vinifera* GN=GSVIVT00023967001 PE=1 SV=1 | | MD01G1177000 | sp|Q40479|ERF2_TOBAC Ethylene-responsive transcription factor 2 OS=*Nicotiana tabacum* GN=ERF2 PE=2 SV=1 | | MD01G1201400 | sp|B0X1Q4|RUMI_CULQU O-glucosyltransferase rumi homolog OS=*Culex quinquefasciatus* GN=CPIJ013394 PE=3 SV=1 | | MD01G1208700 | sp|P27323|HS901_ARATH Heat shock protein 90-1 OS=*Arabidopsis thaliana* GN=HSP90-1 PE=1 SV=3 | | MD01G1213100 | sp|P36908|CHIA_CICAR Acidic endochitinase OS=*Cicer arietinum* PE=2 SV=1 | | MD02G1003800 | sp|Q8S0F0|FH1_ORYSJ Formin-like protein 1 OS=*Oryza sativa* subsp. japonica GN=FH1 PE=2 SV=1 | | MD02G1040000 | sp|O48788|Y2267_ARATH Probable inactive receptor kinase At2g26730 OS=*Arabidopsis thaliana* GN=At2g26730 PE=1 SV=1 | | MD02G1082300 | sp|O82645|IQM1_ARATH IQ domain-containing protein IQM1 OS=*Arabidopsis thaliana* GN=IQM1 PE=1 SV=1 | | MD02G1096100 | sp|Q9LMA8|TI10A_ARATH Protein TIFY 10A OS=*Arabidopsis thaliana* GN=TIFY10A PE=1 SV=1 | | MD02G1121700 | sp|Q9SX98|LHTL8_ARATH Lysine histidine transporter-like 8 OS=*Arabidopsis thaliana* GN=AATL1 PE=1 SV=1 | | MD02G1132200 | sp|Q06942|FL3H_MALDO Naringenin,2-oxoglutarate 3-dioxygenase OS=*Malus domestica* PE=2 SV=1 | | MD02G1205700 | sp|O22150|SAU36_ARATH Auxin-responsive protein SAUR36 OS=*Arabidopsis thaliana* GN=SAUR36 PE=2 SV=1 | | MD02G1244800 | sp|F4HW02|GAT1_ARATH GABA transporter 1 OS=*Arabidopsis thaliana* GN=GAT1 PE=1 SV=1 | | MD03G1001100 | sp|P51091|LDOX_MALDO Leucoanthocyanidin dioxygenase OS=*Malus domestica* GN=ANS PE=2 SV=1 | | MD03G1048000 | sp|Q7XA40|RGA3_SOLBU Putative disease resistance protein RGA3 OS=*Solanum bulbocastanum* GN=RGA3 PE=2 SV=2 | | MD03G1049200 | sp|Q7XA40|RGA3_SOLBU Putative disease resistance protein RGA3 OS=*Solanum bulbocastanum* GN=RGA3 PE=2 SV=2 | | MD03G1071800 | sp|Q8L7Y9|NPC1_ARATH Non-specific phospholipase C1 OS=*Arabidopsis thaliana* GN=NPC1 PE=2 SV=1 | | MD03G1136000 | sp|Q9FK81|Y5258_ARATH Stress-response A/B barrel domain-containing protein At5g22580 OS=*Arabidopsis thaliana* GN=At5g22580 PE=1 SV=1 | | MD03G1286600 | sp|B8B5U8|BAHL2_ORYSI Probable E3 ubiquitin-protein ligase BAH1-like 1 OS=*Oryza sativa* subsp. indica GN=OsI_27296 PE=3 SV=1 | | MD03G1292200 | sp|Q53RH0|ERS1_ORYSJ Probable ethylene response sensor 1 OS=*Oryza sativa* subsp. japonica GN=ERS1 PE=2 SV=1 | | MD04G1003000 | sp|Q9AU11|PKS1_RUBID Polyketide synthase 1 OS=*Rubus idaeus* GN=PKS1 PE=1 SV=1 | | MD04G1009000 | sp|Q9SVQ0|ERF62_ARATH Ethylene-responsive transcription factor ERF062 OS=*Arabidopsis thaliana* GN=ERF062 PE=2 SV=1 | | MD04G1009800 | sp|Q9SFU3|PPA15_ARATH Purple acid phosphatase 15 OS=*Arabidopsis thaliana* GN=PAP15 PE=1 SV=1 | | MD04G1017600 | sp|Q84R16|GT14_ARATH Probable xyloglucan galactosyltransferase GT14 OS=*Arabidopsis thaliana* GN=GT14 PE=2 SV=1 | | MD04G1022400 | sp|Q43207|FKB70_WHEAT 70 kDa peptidyl-prolyl isomerase OS=*Triticum aestivum* GN=FKBP70 PE=1 SV=1 | | MD04G1208600 | sp|Q8RXX9|ATL6_ARATH E3 ubiquitin-protein ligase ATL6 OS=*Arabidopsis thaliana* GN=ATL6 PE=1 SV=2 | | MD04G1212400 | sp|Q940G6|GID1C_ARATH Gibberellin receptor GID1C OS=*Arabidopsis thaliana* GN=GID1C PE=1 SV=1 | | MD04G1225400 | sp|P43082|HEVL_ARATH Hevein-like preproprotein OS=*Arabidopsis thaliana* GN=HEL PE=1 SV=1 | | MD04G1233100 | sp|P93604|LRK10_WHEAT Rust resistance kinase Lr10 OS=*Triticum aestivum* GN=LRK10 PE=2 SV=1 | | MD05G1013200 | sp|P48977|ADH_MALDO Alcohol dehydrogenase OS=*Malus domestica* GN=ADH PE=2 SV=1 | | MD05G1021100 | sp|Q49537|VLPE_MYCHR Variant surface antigen E OS=*Mycoplasma hyorhinis* GN=vlpE PE=4 SV=1 | | MD05G1092300 | sp|O82333|GH31_ARATH Probable indole-3-acetic acid-amido synthetase GH3.1 OS=*Arabidopsis thaliana* GN=GH3.1 PE=2 SV=1 | | MD05G1096400 | sp|B8BB68|BAK1_ORYSI LRR receptor kinase BAK1 OS=*Oryza sativa* subsp. indica GN=BAK1 PE=2 SV=1 | | MD05G1108200 | sp|P10978|POLX_TOBAC Retrovirus-related Pol polyprotein from transposon TNT 1-94 OS=*Nicotiana tabacum* PE=2 SV=1 | | MD05G1143500 | sp|O64967|HMDH2_GOSHI 3-hydroxy-3-methylglutaryl-coenzyme A reductase 2 OS=*Gossypium hirsutum* GN=HMG2 PE=3 SV=1 | | MD05G1158800 | sp|Q9SKX1|IBH1_ARATH Transcription factor IBH1 OS=*Arabidopsis thaliana* GN=IBH1 PE=1 SV=1 | | MD05G1176500 | sp|Q6ICB0|DESI1_HUMAN Desumoylating isopeptidase 1 OS=*Homo sapiens* GN=DESI1 PE=1 SV=1 | | MD05G1192900 | sp|C0QGP2|RLME_DESAH Ribosomal RNA large subunit methyltransferase E OS=*Desulfobacterium autotrophicum* GN=rlmE PE=3 SV=1 | | MD05G1205000 | sp|Q39194|PSBW_ARATH Photosystem II reaction center W protein, chloroplastic OS=*Arabidopsis thaliana* GN=PSBW PE=1 SV=2 | | MD05G1223500 | sp|P46283|S17P_ARATH Sedoheptulose-1,7-bisphosphatase, chloroplastic OS=*Arabidopsis thaliana* GN=At3g55800 PE=2 SV=1 | | MD05G1259100 | sp|Q9SHE9|LBD4_ARATH LOB domain-containing protein 4 OS=*Arabidopsis thaliana* GN=LBD4 PE=2 SV=1 | | MD05G1262600 | sp|I7C6E8|C7A52_PANGI Beta-amyrin 28-oxidase OS=*Panax ginseng* PE=2 SV=1 | | MD05G1262700 | sp|Q50EK0|C16B2_PICSI Cytochrome P450 716B2 OS=*Picea sitchensis* GN=CYP716B2 PE=2 SV=1 | | MD05G1289200 | sp|P27495|CB24_TOBAC Chlorophyll a-b binding protein 40, chloroplastic OS=*Nicotiana tabacum* GN=CAB40 PE=2 SV=1 | | MD05G1325800 | sp|Q7XVM8|TIR1B_ORYSJ Transport inhibitor response 1-like protein Os04g0395600 OS=*Oryza sativa* subsp. japonica GN=Os04g0395600 PE=2 SV=1 | | MD06G1010100 | sp|P54797|TNG2_MOUSE Transport and Golgi organization 2 homolog OS=*Mus musculus* GN=Tango2 PE=1 SV=1 | | MD06G1036900 | sp|Q8WZ42|TITIN_HUMAN Titin OS=*Homo sapiens* GN=TTN PE=1 SV=4 | | MD06G1066000 | sp|Q9SU40|SKU5_ARATH Monocopper oxidase-like protein SKU5 OS=*Arabidopsis thaliana* GN=SKU5 PE=1 SV=1 | | MD06G1071600 | sp|P51091|LDOX_MALDO Leucoanthocyanidin dioxygenase OS=*Malus domestica* GN=ANS PE=2 SV=1 | | MD06G1079100 | sp|Q9SMT7|4CLLA_ARATH Oxalate--CoA ligase OS=*Arabidopsis thaliana* GN=AAE3 PE=1 SV=1 | | MD06G1162100 | sp|P92962|PROT2_ARATH Proline transporter 2 OS=*Arabidopsis thaliana* GN=PROT2 PE=1 SV=1 | | MD06G1182400 | sp|Q9ZWS6|ARR6_ARATH Two-component response regulator ARR6 OS=*Arabidopsis thaliana* GN=ARR6 PE=1 SV=2 | | MD06G1201700 | sp|Q9SBQ9|F3PH_PETHY Flavonoid 3'-monooxygenase OS=*Petunia hybrida* GN=CYP75B2 PE=2 SV=1 | | MD06G1213500 | sp|H2DH18|C7A12_PANGI Cytochrome P450 CYP736A12 OS=*Panax ginseng* PE=2 SV=1 | | MD06G1229100 | sp|Q5QMT0|BGL01_ORYSJ Beta-glucosidase 1 OS=*Oryza sativa* subsp. japonica GN=BGLU1 PE=2 SV=1 | | MD07G1007400 | sp|Q2V6J9|UFOG7_FRAAN UDP-glucose flavonoid 3-O-glucosyltransferase 7 OS=*Fragaria ananassa* GN=GT7 PE=1 SV=1 | | MD07G1053800 | sp|O24606|EIN3_ARATH Protein ETHYLENE INSENSITIVE 3 OS=*Arabidopsis thaliana* GN=EIN3 PE=1 SV=1 | | MD07G1077400 | sp|Q9SRQ7|NPC4_ARATH Non-specific phospholipase C4 OS=*Arabidopsis thaliana* GN=NPC4 PE=1 SV=1 | | MD07G1108600 | sp|Q55DR1|ABCGE_DICDI ABC transporter G family member 14 OS=*Dictyostelium discoideum* GN=abcG14 PE=3 SV=1 | | MD07G1177600 | sp|P37645|YHJG_ECOLI Uncharacterized protein YhjG OS=*Escherichia coli* (strain K12) GN=yhjG PE=4 SV=3 | | MD07G1230500 | sp|A7NY33|PER4_VITVI Peroxidase 4 OS=*Vitis vinifera* GN=GSVIVT00023967001 PE=1 SV=1 | | MD07G1233100 | sp|O22874|EXPA8_ARATH Expansin-A8 OS=*Arabidopsis thaliana* GN=EXPA8 PE=2 SV=1 | | MD07G1283700 | sp|Q9SUQ3|Y4374_ARATH Probable inactive receptor kinase At4g23740 OS=*Arabidopsis thaliana* GN=At4g23740 PE=2 SV=1 | | MD08G1022400 | sp|Q94AP3|WAT1_ARATH Protein WALLS ARE THIN 1 OS=*Arabidopsis thaliana* GN=WAT1 PE=1 SV=1 | | MD08G1091700 | sp|Q9STX3|GID2_ARATH F-box protein GID2 OS=*Arabidopsis thaliana* GN=GID2 PE=1 SV=1 | | MD08G1117300 | sp|P28734|AATC_DAUCA Aspartate aminotransferase, cytoplasmic OS=*Daucus carota* PE=2 SV=1 | | MD08G1163100 | sp|Q9CAP8|LACS9_ARATH Long chain acyl-CoA synthetase 9, chloroplastic OS=*Arabidopsis thaliana* GN=LACS9 PE=1 SV=1 | | MD08G1186100 | sp|Q9ZPE7|EXO_ARATH Protein EXORDIUM OS=*Arabidopsis thaliana* GN=EXO PE=2 SV=1 | | MD08G1187200 | sp|Q0D4J7|SAPK2_ORYSJ Serine/threonine-protein kinase SAPK2 OS=*Oryza sativa* subsp. japonica GN=SAPK2 PE=1 SV=1 | | MD09G1008800 | sp|Q9FYA2|WRK75_ARATH Probable WRKY transcription factor 75 OS=*Arabidopsis thaliana* GN=WRKY75 PE=2 SV=1 | | MD09G1009100 | sp|O22328|AGL8_SOLCO Agamous-like MADS-box protein AGL8 homolog OS=*Solanum commersonii* GN=SCM1 PE=2 SV=1 | | MD09G1032100 | sp|Q9LK90|ALA8_ARATH Probable phospholipid-transporting ATPase 8 OS=*Arabidopsis thaliana* GN=ALA8 PE=3 SV=1 | | MD09G1051000 | sp|P81392|MYB06_ANTMA Myb-related protein 306 OS=*Antirrhinum majus* GN=MYB306 PE=2 SV=1 | | MD09G1052400 | sp|P0C8Q9|Y5902_ARATH Uncharacterized protein At5g19025 OS=*Arabidopsis thaliana* GN=At5g19025 PE=2 SV=3 | | MD09G1091000 | sp|Q6I581|GH35_ORYSJ Jasmonic acid-amido synthetase JAR1 OS=*Oryza sativa* subsp. japonica GN=GH3.5 PE=2 SV=1 | | MD09G1137100 | sp|Q9SA77|ARAE1_ARATH UDP-arabinose 4-epimerase 1 OS=*Arabidopsis thaliana* GN=MUR4 PE=1 SV=1 | | MD09G1152600 | sp|P35694|XTH2_SOYBN Xyloglucan endotransglucosylase/hydrolase 2 OS=*Glycine max* PE=2 SV=1 | | MD09G1183000 | sp|Q4VYC8|NSP1_MEDTR Nodulation-signaling pathway 1 protein OS=*Medicago truncatula* GN=NSP1 PE=1 SV=1 | | MD09G1208000 | sp|Q38826|IAA8_ARATH Auxin-responsive protein IAA8 OS=*Arabidopsis thaliana* GN=IAA8 PE=1 SV=1 | | MD09G1211600 | sp|Q9ZNR6|PDX12_ARATH Pyridoxal 5'-phosphate synthase-like subunit PDX1.2 OS=*Arabidopsis thaliana* GN=PDX12 PE=1 SV=1 | | MD09G1216100 | sp|P93830|IAA17_ARATH Auxin-responsive protein IAA17 OS=*Arabidopsis thaliana* GN=IAA17 PE=1 SV=2 | | MD09G1251600 | sp|P34913|HYES_HUMAN Bifunctional epoxide hydrolase 2 OS=*Homo sapiens* GN=EPHX2 PE=1 SV=2 | | MD10G1033300 | sp|Q96520|PER12_ARATH Peroxidase 12 OS=*Arabidopsis thaliana* GN=PER12 PE=1 SV=1 | | MD10G1059600 | sp|P32295|ARG7_VIGRR Indole-3-acetic acid-induced protein ARG7 OS=*Vigna radiata* var. radiata GN=ARG7 PE=2 SV=1 | | MD10G1061400 | sp|O65695|SAU50_ARATH Auxin-responsive protein SAUR50 OS=*Arabidopsis thaliana* GN=SAUR50 PE=1 SV=1 | | MD10G1099200 | sp|Q8L7W8|FUCO2_ARATH Alpha-L-fucosidase 2 OS=*Arabidopsis thaliana* GN=FUC95A PE=1 SV=1 | | MD10G1121900 | sp|P83332|TLP1_PRUPE Thaumatin-like protein 1 OS=*Prunus persica* PE=2 SV=1 | | MD10G1193000 | sp|P33077|IAA4_ARATH Auxin-responsive protein IAA4 OS=*Arabidopsis thaliana* GN=IAA4 PE=1 SV=2 | | MD10G1240500 | sp|Q50EK1|C16B1_PICSI Cytochrome P450 716B1 OS=*Picea sitchensis* GN=CYP716B1 PE=2 SV=1 | | MD10G1265400 | sp|P08221|CB21_CUCSA Chlorophyll a-b binding protein of LHCII type I, chloroplastic (Fragment) OS=*Cucumis sativus* PE=2 SV=1 | | MD10G1268900 | sp|O64967|HMDH2_GOSHI 3-hydroxy-3-methylglutaryl-coenzyme A reductase 2 OS=*Gossypium hirsutum* GN=HMG2 PE=3 SV=1 | | MD10G1289200 | sp|P59910|DJB13_HUMAN DnaJ homolog subfamily B member 13 OS=*Homo sapiens* GN=DNAJB13 PE=1 SV=1 | | MD10G1303600 | sp|F4JJJ3|NDB3_ARATH External alternative NAD(P)H-ubiquinone oxidoreductase B3, mitochondrial OS=*Arabidopsis thaliana* GN=NDB3 PE=2 SV=1 | | MD10G1316100 | sp|Q94B38|GPT2_ARATH Glucose-6-phosphate/phosphate translocator 2, chloroplastic OS=*Arabidopsis thaliana* GN=GPT2 PE=2 SV=2 | | MD10G1340100 | sp|P49351|FPPS1_LUPAL Farnesyl pyrophosphate synthase 1 OS=*Lupinus albus* GN=FPS1 PE=2 SV=1 | | MD11G1062500 | sp|P10978|POLX_TOBAC Retrovirus-related Pol polyprotein from transposon TNT 1-94 OS=*Nicotiana tabacum* PE=2 SV=1 | | MD11G1070200 | sp|Q54R82|MKKA_DICDI Mitogen-activated protein kinase kinase kinase A OS=*Dictyostelium discoideum* GN=mkkA PE=1 SV=2 | | MD11G1132700 | sp|Q94KB7|MLO6_ARATH MLO-like protein 6 OS=*Arabidopsis thaliana* GN=MLO6 PE=2 SV=2 | | MD11G1134500 | sp|Q9FKJ0|FK132_ARATH F-box/kelch-repeat protein At5g60570 OS=*Arabidopsis thaliana* GN=At5g60570 PE=2 SV=1 | | MD11G1235400 | sp|Q1ECE0|VAP41_ARATH Vesicle-associated protein 4-1 OS=*Arabidopsis thaliana* GN=PVA41 PE=2 SV=1 | | MD11G1267900 | sp|C0LGN2|Y3148_ARATH Probable leucine-rich repeat receptor-like serine/threonine-protein kinase At3g14840 OS=*Arabidopsis thaliana* GN=LRR-RLK PE=2 SV=1 | | MD11G1306500 | sp|O22259|ERF71_ARATH Ethylene-responsive transcription factor ERF071 OS=*Arabidopsis thaliana* GN=ERF071 PE=2 SV=1 | | MD11G1308400 | sp|Q93WF6|SAG21_ARATH Protein SENESCENCE-ASSOCIATED GENE 21, mitochondrial OS=*Arabidopsis thaliana* GN=SAG21 PE=2 SV=1 | | MD12G1038700 | sp|Q9SRN0|NHL1_ARATH NDR1/HIN1-like protein 1 OS=*Arabidopsis thaliana* GN=NHL1 PE=2 SV=1 | | MD12G1060200 | sp|A3C057|SPL17_ORYSJ Squamosa promoter-binding-like protein 17 OS=*Oryza sativa* subsp. japonica GN=SPL17 PE=2 SV=2 | | MD12G1103500 | sp|Q6T1F5|COMT1_AMMMJ Caffeic acid 3-O-methyltransferase OS=*Ammi majus* GN=COMT PE=1 SV=1 | | MD12G1162400 | sp|Q8L884|LAX4_MEDTR Auxin transporter-like protein 4 OS=*Medicago truncatula* GN=LAX4 PE=2 SV=1 | | MD12G1185400 | sp|O22932|CIPKB_ARATH CBL-interacting serine/threonine-protein kinase 11 OS=*Arabidopsis thaliana* GN=CIPK11 PE=1 SV=1 | | MD12G1198600 | sp|Q8LPH6|MYB86_ARATH Transcription factor MYB86 OS=*Arabidopsis thaliana* GN=MYB86 PE=2 SV=1 | | MD12G1226800 | sp|Q9SAZ5|AHP3_ARATH Histidine-containing phosphotransfer protein 3 OS=*Arabidopsis thaliana* GN=AHP3 PE=1 SV=2 | | MD13G1063100 | sp|Q5R7C5|ERLN2_PONAB Erlin-2 OS=*Pongo abelii* GN=ERLIN2 PE=2 SV=1 | | MD13G1096800 | sp|Q9AWA5|GWD1_SOLTU Alpha-glucan water dikinase, chloroplastic OS=*Solanum tuberosum* GN=R1 PE=1 SV=2 | | MD13G1116100 | sp|Q8VWZ7|C76B6_CATRO Geraniol 8-hydroxylase OS=*Catharanthus roseus* GN=CYP76B6 PE=1 SV=1 | | MD13G1120300 | sp|Q9S840|SPL2_ARATH Squamosa promoter-binding-like protein 2 OS=*Arabidopsis thaliana* GN=SPL2 PE=2 SV=1 | | MD13G1123300 | sp|O65373|BAG5_ARATH BAG family molecular chaperone regulator 5, mitochondrial OS=*Arabidopsis thaliana* GN=BAG5 PE=1 SV=1 | | MD13G1184700 | sp|Q9LE22|CML27_ARATH Probable calcium-binding protein CML27 OS=*Arabidopsis thaliana* GN=CML27 PE=1 SV=1 | | MD13G1222200 | sp|Q38831|IAA13_ARATH Auxin-responsive protein IAA13 OS=*Arabidopsis thaliana* GN=IAA13 PE=1 SV=2 | | MD13G1274600 | sp|Q9M0B6|GAE1_ARATH UDP-glucuronate 4-epimerase 1 OS=*Arabidopsis thaliana* GN=GAE1 PE=1 SV=1 | | MD13G1285100 | sp|Q9AU11|PKS1_RUBID Polyketide synthase 1 OS=*Rubus idaeus* GN=PKS1 PE=1 SV=1 | | MD14G1032700 | sp|P54968|ILR1_ARATH IAA-amino acid hydrolase ILR1 OS=*Arabidopsis thaliana* GN=ILR1 PE=1 SV=2 | | MD14G1037300 | sp|Q9FNH6|NHL3_ARATH NDR1/HIN1-like protein 3 OS=*Arabidopsis thaliana* GN=NHL3 PE=1 SV=1 | | MD14G1041600 | sp|Q8RX29|ATL70_ARATH RING-H2 finger protein ATL70 OS=*Arabidopsis thaliana* GN=ATL70 PE=2 SV=1 | | MD14G1080100 | sp|P52408|E13B_PRUPE Glucan endo-1,3-beta-glucosidase, basic isoform OS=*Prunus persica* GN=GNS1 PE=3 SV=1 | | MD14G1102200 | sp|Q9SMT7|4CLLA_ARATH Oxalate--CoA ligase OS=*Arabidopsis thaliana* GN=AAE3 PE=1 SV=1 | | MD15G1024100 | sp|Q9XES5|DFRA_MALDO Bifunctional dihydroflavonol 4-reductase/flavanone 4-reductase OS=*Malus domestica* GN=DFR PE=1 SV=1 | | MD15G1075800 | sp|Q9STX3|GID2_ARATH F-box protein GID2 OS=*Arabidopsis thaliana* GN=GID2 PE=1 SV=1 | | MD15G1090600 | sp|Q8LAL2|IAA26_ARATH Auxin-responsive protein IAA26 OS=*Arabidopsis thaliana* GN=IAA26 PE=1 SV=2 | | MD15G1191800 | sp|Q38826|IAA8_ARATH Auxin-responsive protein IAA8 OS=*Arabidopsis thaliana* GN=IAA8 PE=1 SV=1 | | MD15G1246200 | sp|Q06942|FL3H_MALDO Naringenin,2-oxoglutarate 3-dioxygenase OS=*Malus domestica* PE=2 SV=1 | | MD15G1436500 | sp|O48922|C98A2_SOYBN Cytochrome P450 98A2 OS=*Glycine max* GN=CYP98A2 PE=2 SV=1 | | MD16G1140800 | sp|Q9LYU3|EF113_ARATH Ethylene-responsive transcription factor ERF113 OS=*Arabidopsis thaliana* GN=ERF113 PE=2 SV=1 | | MD17G1042500 | sp|Q9LHD2|GATLA_ARATH Probable galacturonosyltransferase-like 10 OS=*Arabidopsis thaliana* GN=GATL10 PE=2 SV=1 | | MD17G1189100 | sp|Q38826|IAA8_ARATH Auxin-responsive protein IAA8 OS=*Arabidopsis thaliana* GN=IAA8 PE=1 SV=1 | | MD17G1209000 | sp|Q9XI33|WIN1_ARATH Ethylene-responsive transcription factor WIN1 OS=Arabidopsis thaliana GN=WIN1 PE=2 SV=1 |  | **Table S3.** List of genes related to ‘Signal transduction mechanisms’ from the MElightcyan module | | | | --- | --- | --- | | **ID** | **KOG_class_annotation** | **NR_annotation** | | MD03G1292200 | Signal transduction mechanisms | probable ethylene response sensor 1 [*Malus* *domestica*] | | MD06G1001100 | Signal transduction mechanisms | ethylene receptor [*Malus* *domestica*] | | MD08G1200300 | Signal transduction mechanisms | PREDICTED: serine/threonine-protein kinase CTR1-like [*Malus* *domestica*] | | MD13G1209700 | Signal transduction mechanisms | PREDICTED: ethylene receptor 2-like [*Malus* *domestica*] | | MD15G1387500 | Signal transduction mechanisms | PREDICTED: serine/threonine-protein kinase CTR1 isoform X1 [*Pyrus* x *bretschneideri*] | | MD16G1212500 | Signal transduction mechanisms | PREDICTED: ethylene receptor 2-like [*Malus* *domestica*] | | MD01G1069300 | Signal transduction mechanisms | PREDICTED: probable serine/threonine-protein kinase At1g01540 [*Pyrus* x *bretschneideri*] | | MD01G1091000 | Signal transduction mechanisms | PREDICTED: serine/threonine-protein kinase ATG1a-like isoform X2 [*Malus* *domestica*] | | MD01G1114700 | Signal transduction mechanisms | PREDICTED: serine/threonine-protein kinase CDL1-like [*Malus* *domestica*] | | MD01G1139200 | Signal transduction mechanisms | PREDICTED: protein phosphatase 2C 37-like [*Pyrus* x *bretschneideri*] | | MD01G1195700 | Signal transduction mechanisms | PREDICTED: calcium-dependent protein kinase 26-like [*Malus* *domestica*] | | MD02G1061700 | Signal transduction mechanisms | PREDICTED: serine/threonine protein phosphatase 2A 57 kDa regulatory subunit B&apos; iota isoform-like [*Pyrus* x *bretschneideri*] | | MD02G1076100 | Signal transduction mechanisms | PREDICTED: inactive protein kinase SELMODRAFT_444075-like isoform X2 [*Malus* *domestica*] | | MD02G1079800 | Signal transduction mechanisms | PREDICTED: proline-rich protein 36 isoform X5 [*Pyrus* x *bretschneideri*] | | MD02G1114500 | Signal transduction mechanisms | PREDICTED: SRSF protein kinase 1-like [*Malus* *domestica*] | | MD02G1159100 | Signal transduction mechanisms | PREDICTED: wall-associated receptor kinase-like 14 [*Malus* *domestica*] | | MD02G1174000 | Signal transduction mechanisms | PREDICTED: probable serine/threonine-protein kinase At1g01540 [*Malus* *domestica*] | | MD02G1198700 | Signal transduction mechanisms | PREDICTED: LOW QUALITY PROTEIN: receptor-like serine/threonine-protein kinase At2g45590, partial [*Malus* *domestica*] | | MD02G1222400 | Signal transduction mechanisms | PREDICTED: probable receptor-like protein kinase At5g47070 [*Malus* *domestica*] | | MD02G1246100 | Signal transduction mechanisms | hypothetical protein PRUPE_2G088400 [Prunus persica] | | MD02G1274000 | Signal transduction mechanisms | PREDICTED: LEAF RUST 10 DISEASE-RESISTANCE LOCUS RECEPTOR-LIKE PROTEIN KINASE-like 2.1 [*Pyrus* x *bretschneideri*] | | MD03G1002700 | Signal transduction mechanisms | PREDICTED: probable receptor-like protein kinase At5g56460 [*Pyrus* x *bretschneideri*] | | MD03G1054700 | Signal transduction mechanisms | PREDICTED: CBL-interacting serine/threonine-protein kinase 11-like [*Malus* *domestica*] | | MD03G1059600 | Signal transduction mechanisms | CBL-interacting serine/threonine-protein kinase 10-like [*Malus* *domestica*] | | MD03G1062000 | Signal transduction mechanisms | PREDICTED: probable LIM domain-containing serine/threonine-protein kinase DDB_G0287001 [*Malus* *domestica*] | | MD03G1062100 | Signal transduction mechanisms | PREDICTED: probable LIM domain-containing serine/threonine-protein kinase DDB_G0287001 [*Malus* *domestica*] | | MD03G1065600 | Signal transduction mechanisms | PREDICTED: probable protein phosphatase 2C 25 [*Malus* *domestica*] | | MD03G1077700 | Signal transduction mechanisms | PREDICTED: calmodulin-binding receptor-like cytoplasmic kinase 2 [*Malus* *domestica*] | | MD03G1092200 | Signal transduction mechanisms | PREDICTED: protein kinase APK1A, chloroplastic [*Malus* *domestica*] | | MD03G1107700 | Signal transduction mechanisms | PREDICTED: mitogen-activated protein kinase homolog NTF6-like [*Malus* *domestica*] | | MD03G1120900 | Signal transduction mechanisms | PREDICTED: receptor-like serine/threonine-protein kinase NCRK isoform X2 [*Malus* *domestica*] | | MD03G1161500 | Signal transduction mechanisms | PREDICTED: uncharacterized protein LOC103425540 [*Malus* *domestica*] | | MD03G1221300 | Signal transduction mechanisms | PREDICTED: receptor-like protein kinase THESEUS 1 [*Pyrus* x *bretschneideri*] | | MD03G1272700 | Signal transduction mechanisms | PREDICTED: receptor-like cytosolic serine/threonine-protein kinase RBK2 [*Malus* *domestica*] | | MD04G1008500 | Signal transduction mechanisms | PREDICTED: probable calcium-binding protein CML22 isoform X1 [*Malus* *domestica*] | | MD04G1008600 | Signal transduction mechanisms | PREDICTED: receptor like protein kinase S.2 [*Malus* *domestica*] | | MD04G1083500 | Signal transduction mechanisms | PREDICTED: serine/threonine-protein phosphatase PP1 isoform X2 [*Pyrus* x *bretschneideri*] | | MD04G1154500 | Signal transduction mechanisms | PREDICTED: calcium-dependent protein kinase 13 [*Malus* *domestica*] | | MD04G1180400 | Signal transduction mechanisms | PREDICTED: serine/threonine-protein kinase At5g01020-like [*Malus* *domestica*] | | MD04G1197100 | Signal transduction mechanisms | PREDICTED: probable protein phosphatase 2C 34 [*Malus* *domestica*] | | MD04G1208400 | Signal transduction mechanisms | PREDICTED: serine/threonine-protein kinase CDL1-like [*Pyrus* x *bretschneideri*] | | MD04G1229200 | Signal transduction mechanisms | PREDICTED: phosphoenolpyruvate carboxylase kinase 1-like [*Pyrus* x *bretschneideri*] | | MD05G1041800 | Signal transduction mechanisms | PREDICTED: calcium-dependent protein kinase 4-like [*Malus* *domestica*] | | MD05G1075300 | Signal transduction mechanisms | CDPK-related kinase 5-like [Prunus avium] | | MD05G1082700 | Signal transduction mechanisms | PREDICTED: probable receptor-like protein kinase At1g49730 isoform X1 [*Pyrus* x *bretschneideri*] | | MD05G1125100 | Signal transduction mechanisms | PREDICTED: serine/threonine protein phosphatase 2A 57 kDa regulatory subunit B&apos; beta isoform-like [*Malus* *domestica*] | | MD05G1185400 | Signal transduction mechanisms | PREDICTED: receptor-like serine/threonine-protein kinase ALE2 isoform X2 [*Malus* *domestica*] | | MD06G1006700 | Signal transduction mechanisms | PREDICTED: probable protein phosphatase 2C 27 [*Malus* *domestica*] | | MD06G1044400 | Signal transduction mechanisms | PREDICTED: probable receptor-like protein kinase At5g47070 [*Pyrus* x *bretschneideri*] | | MD07G1093200 | Signal transduction mechanisms | PREDICTED: probable receptor-like protein kinase At5g47070 [*Malus* *domestica*] | | MD07G1151100 | Signal transduction mechanisms | PREDICTED: probable calcium-binding protein CML36 [*Malus* *domestica*] | | MD07G1182700 | Signal transduction mechanisms | PREDICTED: serine/threonine-protein kinase CDL1-like [*Pyrus* x *bretschneideri*] | | MD07G1191300 | Signal transduction mechanisms | PREDICTED: receptor-like protein kinase THESEUS 1 [*Malus* *domestica*] | | MD07G1193800 | Signal transduction mechanisms | PREDICTED: probable protein phosphatase 2C 78 [*Malus* *domestica*] | | MD07G1203700 | Signal transduction mechanisms | PREDICTED: protein phosphatase 2C 37 [*Malus* *domestica*] | | MD07G1288800 | Signal transduction mechanisms | PREDICTED: calcineurin B-like protein 4 isoform X2 [*Malus* *domestica*] | | MD07G1291000 | Signal transduction mechanisms | PREDICTED: probable protein phosphatase 2C 51 [*Pyrus* x *bretschneideri*] | | MD07G1293900 | Signal transduction mechanisms | PREDICTED: protein phosphatase 2C 57-like [*Malus* *domestica*] | | MD08G1038900 | Signal transduction mechanisms | PREDICTED: wall-associated receptor kinase-like 14 [*Malus* *domestica*] | | MD08G1066300 | Signal transduction mechanisms | PREDICTED: protein phosphatase 2C 77-like [*Malus* *domestica*] | | MD08G1097900 | Signal transduction mechanisms | PREDICTED: probable serine/threonine-protein kinase At1g01540 [*Malus* *domestica*] | | MD08G1102700 | Signal transduction mechanisms | PREDICTED: probable protein phosphatase 2C 12 [*Pyrus* x *bretschneideri*] | | MD08G1137200 | Signal transduction mechanisms | PREDICTED: serine/threonine protein phosphatase 2A 57 kDa regulatory subunit B&apos; iota isoform-like [*Malus* *domestica*] | | MD09G1024000 | Signal transduction mechanisms | PREDICTED: serine/threonine-protein kinase HT1-like [*Pyrus* x *bretschneideri*] | | MD09G1069400 | Signal transduction mechanisms | PREDICTED: receptor-like protein kinase FERONIA [*Malus* *domestica*] | | MD09G1121900 | Signal transduction mechanisms | CHASE histidine kinase 2 [*Malus* *domestica*] | | MD09G1155800 | Signal transduction mechanisms | PREDICTED: serine/threonine-protein kinase TNNI3K [*Malus* *domestica*] | | MD09G1165900 | Signal transduction mechanisms | PREDICTED: mitogen-activated protein kinase kinase kinase YODA-like [*Malus* *domestica*] | | MD09G1193100 | Signal transduction mechanisms | PREDICTED: receptor-like serine/threonine-protein kinase ALE2 [*Malus* *domestica*] | | MD09G1194900 | Signal transduction mechanisms | PREDICTED: inactive protein kinase SELMODRAFT_444075 isoform X2 [*Malus* *domestica*] | | MD09G1262600 | Signal transduction mechanisms | PREDICTED: protein kinase APK1B, chloroplastic-like [*Malus* *domestica*] | | MD09G1262900 | Signal transduction mechanisms | PREDICTED: probable calcium-binding protein CML23 [*Malus* *domestica*] | | MD09G1287400 | Signal transduction mechanisms | PREDICTED: protein-tyrosine-phosphatase PTP1-like [*Malus* *domestica*] | | MD10G1089900 | Signal transduction mechanisms | PREDICTED: CDPK-related kinase 5-like [*Malus* *domestica*] | | MD10G1173600 | Signal transduction mechanisms | PREDICTED: receptor-like serine/threonine-protein kinase ALE2 isoform X3 [*Malus* *domestica*] | | MD10G1278300 | Signal transduction mechanisms | CBL-interacting serine/threonine-protein kinase 19 [*Malus* *domestica*] | | MD10G1293400 | Signal transduction mechanisms | PREDICTED: serine/threonine-protein kinase HT1-like [*Malus* *domestica*] | | MD10G1326000 | Signal transduction mechanisms | PREDICTED: protein STRUBBELIG-RECEPTOR FAMILY 8 [*Malus* *domestica*] | | MD11G1056100 | Signal transduction mechanisms | CBL-interacting serine/threonine-protein kinase 11-like [*Malus* *domestica*] | | MD11G1056600 | Signal transduction mechanisms | PREDICTED: probable protein phosphatase 2C 26 [*Malus* *domestica*] | | MD11G1065600 | Signal transduction mechanisms | PREDICTED: probable LIM domain-containing serine/threonine-protein kinase DDB_G0287001 [*Malus* *domestica*] | | MD11G1070500 | Signal transduction mechanisms | PREDICTED: probable protein phosphatase 2C 25 [*Pyrus* x *bretschneideri*] | | MD11G1135900 | Signal transduction mechanisms | PREDICTED: protein kinase APK1A, chloroplastic-like isoform X1 [*Malus* *domestica*] | | MD11G1273500 | Signal transduction mechanisms | PREDICTED: receptor-like serine/threonine-protein kinase At1g78530 [*Malus* *domestica*] | | MD12G1015200 | Signal transduction mechanisms | PREDICTED: C-type lectin receptor-like tyrosine-protein kinase At1g52310 [*Malus* *domestica*] | | MD12G1087300 | Signal transduction mechanisms | PREDICTED: probable calcium-binding protein CML36 [*Malus* *domestica*] | | MD12G1129700 | Signal transduction mechanisms | PREDICTED: probable protein phosphatase 2C 38 [*Malus* *domestica*] | | MD12G1185400 | Signal transduction mechanisms | CBL-interacting serine/threonine-protein kinase 14 [*Malus* *domestica*] | | MD12G1195800 | Signal transduction mechanisms | PREDICTED: serine/threonine-protein kinase At5g01020-like [*Malus* *domestica*] | | MD12G1210000 | Signal transduction mechanisms | PREDICTED: probable protein phosphatase 2C 34 [*Malus* *domestica*] | | MD12G1226800 | Signal transduction mechanisms | PREDICTED: histidine-containing phosphotransfer protein 1 [*Malus* *domestica*] | | MD13G1017000 | Signal transduction mechanisms | PREDICTED: histidine kinase 4 [*Malus* *domestica*] | | MD13G1050700 | Signal transduction mechanisms | PREDICTED: probable calcium-binding protein CML16 [*Pyrus* x *bretschneideri*] | | MD13G1050900 | Signal transduction mechanisms | PREDICTED: proline-rich receptor-like protein kinase PERK9 isoform X2 [*Pyrus* x *bretschneideri*] | | MD13G1081800 | Signal transduction mechanisms | PREDICTED: LEAF RUST 10 DISEASE-RESISTANCE LOCUS RECEPTOR-LIKE PROTEIN KINASE-like 1.5 [*Malus* *domestica*] | | MD13G1088100 | Signal transduction mechanisms | PREDICTED: serine/threonine-protein kinase CDL1-like [*Malus* *domestica*] | | MD13G1103900 | Signal transduction mechanisms | PREDICTED: serine/threonine-protein kinase AFC1-like [*Malus* *domestica*] | | MD13G1129700 | Signal transduction mechanisms | PREDICTED: inactive leucine-rich repeat receptor-like protein kinase CORYNE [*Malus* *domestica*] | | MD13G1141100 | Signal transduction mechanisms | PREDICTED: probable receptor-like protein kinase At5g18500 [*Malus* *domestica*] | | MD13G1141200 | Signal transduction mechanisms | PREDICTED: pto-interacting protein 1 [*Malus* *domestica*] | | MD13G1169100 | Signal transduction mechanisms | PREDICTED: probable protein phosphatase 2C 40 [*Malus* *domestica*] | | MD13G1176300 | Signal transduction mechanisms | histidine-containing phosphotransfer 1a [*Malus* *domestica*] | | MD13G1176500 | Signal transduction mechanisms | PREDICTED: receptor-like cytosolic serine/threonine-protein kinase RBK2 isoform X1 [*Malus* *domestica*] | | MD13G1231900 | Signal transduction mechanisms | PREDICTED: serine/threonine-protein kinase At5g01020-like isoform X2 [*Pyrus* x *bretschneideri*] | | MD13G1265500 | Signal transduction mechanisms | CBL-interacting serine/threonine-protein kinase 21 [*Malus* *domestica*] | | MD14G1083200 | Signal transduction mechanisms | PREDICTED: LOW QUALITY PROTEIN: probable calcium-binding protein CML36 [*Malus* *domestica*] | | MD15G1006000 | Signal transduction mechanisms | PREDICTED: serine/threonine-protein kinase STY46-like [*Malus* *domestica*] | | MD15G1041900 | Signal transduction mechanisms | PREDICTED: probable protein phosphatase 2C 60 [*Malus* *domestica*] | | MD15G1080000 | Signal transduction mechanisms | PREDICTED: probable serine/threonine-protein kinase At1g01540 [*Malus* *domestica*] | | MD15G1115800 | Signal transduction mechanisms | PREDICTED: serine/threonine protein phosphatase 2A 57 kDa regulatory subunit B&apos; iota isoform-like [*Malus* *domestica*] | | MD15G1131500 | Signal transduction mechanisms | PREDICTED: probable receptor-like protein kinase At1g80640 isoform X1 [*Malus* *domestica*] | | MD15G1168500 | Signal transduction mechanisms | PREDICTED: probable receptor-like protein kinase At5g15080 [*Malus* *domestica*] | | MD15G1184000 | Signal transduction mechanisms | PREDICTED: LOW QUALITY PROTEIN: proline-rich receptor-like protein kinase PERK1 [*Malus* *domestica*] | | MD15G1207500 | Signal transduction mechanisms | PREDICTED: receptor-like serine/threonine-protein kinase ALE2 [*Malus* *domestica*] | | MD15G1229800 | Signal transduction mechanisms | PREDICTED: probable protein phosphatase 2C 47 [*Pyrus* x *bretschneideri*] | | MD15G1243500 | Signal transduction mechanisms | histidine kinase 1-like [*Malus* *domestica*] | | MD15G1289500 | Signal transduction mechanisms | PREDICTED: probable receptor-like protein kinase At2g23200 [*Malus* *domestica*] | | MD15G1321000 | Signal transduction mechanisms | serine/threonine-protein kinase SAPK2-like [*Malus* *domestica*] | | MD15G1381000 | Signal transduction mechanisms | PREDICTED: serine/threonine-protein kinase-like protein At1g28390 [*Malus* *domestica*] | | MD16G1051200 | Signal transduction mechanisms | PREDICTED: probable calcium-binding protein CML16 [*Malus* *domestica*] | | MD16G1051300 | Signal transduction mechanisms | PREDICTED: LOW QUALITY PROTEIN: proline-rich receptor-like protein kinase PERK9 [*Malus* *domestica*] | | MD16G1130000 | Signal transduction mechanisms | PREDICTED: inactive leucine-rich repeat receptor-like protein kinase CORYNE [*Malus* *domestica*] | | MD16G1136800 | Signal transduction mechanisms | PREDICTED: pyruvate dehydrogenase (acetyl-transferring) kinase, mitochondrial-like isoform X2 [*Malus* *domestica*] | | MD16G1137200 | Signal transduction mechanisms | PREDICTED: probable receptor-like protein kinase At5g18500 [*Malus* *domestica*] | | MD16G1173100 | Signal transduction mechanisms | PREDICTED: probable receptor-like protein kinase At2g39360 isoform X1 [*Malus* *domestica*] | | MD16G1236700 | Signal transduction mechanisms | PREDICTED: serine/threonine-protein kinase At5g01020-like [*Pyrus* x *bretschneideri*] | | MD16G1256200 | Signal transduction mechanisms | PREDICTED: probable receptor-like serine/threonine-protein kinase At5g57670 [*Malus* *domestica*] | | MD16G1277400 | Signal transduction mechanisms | PREDICTED: protein phosphatase 2C 29-like [*Malus* *domestica*] | | MD17G1072800 | Signal transduction mechanisms | PREDICTED: proline-rich receptor-like protein kinase PERK8 [*Malus* *domestica*] | | MD17G1079800 | Signal transduction mechanisms | PREDICTED: probable protein phosphatase 2C 52 [*Malus* *domestica*] | | MD17G1113000 | Signal transduction mechanisms | PREDICTED: histidine kinase 2 [*Malus* *domestica*] | | MD17G1138000 | Signal transduction mechanisms | PREDICTED: probable receptor-like protein kinase At2g42960 [*Malus* *domestica*] | | MD17G1223700 | Signal transduction mechanisms | PREDICTED: putative receptor-like protein kinase At1g80870 [*Malus* *domestica*] | | MD17G1247800 | Signal transduction mechanisms | PREDICTED: LEAF RUST 10 DISEASE-RESISTANCE LOCUS RECEPTOR-LIKE PROTEIN KINASE-like 1.3 isoform X2 [Prunus mume] | | MD17G1257900 | Signal transduction mechanisms | PREDICTED: probable calcium-binding protein CML23 [*Malus* *domestica*] | | MD17G1265400 | Signal transduction mechanisms | PREDICTED: serine/threonine-protein kinase CDL1 [*Malus* *domestica*] | | MD17G1286800 | Signal transduction mechanisms | PREDICTED: ALA-interacting subunit 5-like [*Malus* *domestica*] | |
| --- | --- | --- | --- | --- | --- | --- | --- | --- | --- | --- | --- | --- | --- | --- | --- | --- | --- | --- | --- | --- | --- | --- | --- | --- | --- | --- | --- | --- | --- | --- | --- | --- | --- | --- | --- | --- | --- | --- | --- | --- | --- | --- | --- | --- | --- | --- | --- | --- | --- | --- | --- | --- | --- | --- | --- | --- | --- | --- | --- | --- | --- | --- | --- | --- | --- | --- | --- | --- | --- | --- | --- | --- | --- | --- | --- | --- | --- | --- | --- | --- | --- | --- | --- | --- | --- | --- | --- | --- | --- | --- | --- | --- | --- | --- | --- | --- | --- | --- | --- | --- | --- | --- | --- | --- | --- | --- | --- | --- | --- | --- | --- | --- | --- | --- | --- | --- | --- | --- | --- | --- | --- | --- | --- | --- | --- | --- | --- | --- | --- | --- | --- | --- | --- | --- | --- | --- | --- | --- | --- | --- | --- | --- | --- | --- | --- | --- | --- | --- | --- | --- | --- | --- | --- | --- | --- | --- | --- | --- | --- | --- | --- | --- | --- | --- | --- | --- | --- | --- | --- | --- | --- | --- | --- | --- | --- | --- | --- | --- | --- | --- | --- | --- | --- | --- | --- | --- | --- | --- | --- | --- | --- | --- | --- | --- | --- | --- | --- | --- | --- | --- | --- | --- | --- | --- | --- | --- | --- | --- | --- | --- | --- | --- | --- | --- | --- | --- | --- | --- | --- | --- | --- | --- | --- | --- | --- | --- | --- | --- | --- | --- | --- | --- | --- | --- | --- | --- | --- | --- | --- | --- | --- | --- | --- | --- | --- | --- | --- | --- | --- | --- | --- | --- | --- | --- | --- | --- | --- | --- | --- | --- | --- | --- | --- | --- | --- | --- | --- | --- | --- | --- | --- | --- | --- | --- | --- | --- | --- | --- | --- | --- | --- | --- | --- | --- | --- | --- | --- | --- | --- | --- | --- | --- | --- | --- | --- | --- | --- | --- | --- | --- | --- | --- | --- | --- | --- | --- | --- | --- | --- | --- | --- | --- | --- | --- | --- | --- | --- | --- | --- | --- | --- | --- | --- | --- | --- | --- | --- | --- | --- | --- | --- | --- | --- | --- | --- | --- | --- | --- | --- | --- | --- | --- | --- | --- | --- | --- | --- | --- | --- | --- | --- | --- | --- | --- | --- | --- | --- | --- | --- | --- | --- | --- | --- | --- | --- | --- | --- | --- | --- | --- | --- | --- | --- | --- | --- | --- | --- | --- | --- | --- | --- | --- | --- | --- | --- | --- | --- | --- | --- | --- | --- | --- | --- | --- | --- | --- | --- | --- | --- | --- | --- | --- | --- | --- | --- | --- | --- | --- | --- | --- | --- | --- | --- | --- | --- | --- | --- | --- | --- | --- | --- | --- | --- | --- | --- | --- | --- | --- | --- | --- | --- | --- | --- | --- | --- | --- | --- | --- | --- | --- | --- | --- | --- | --- | --- | --- | --- | --- | --- | --- | --- | --- | --- | --- | --- | --- | --- | --- | --- | --- | --- | --- | --- | --- | --- | --- | --- | --- | --- | --- | --- | --- | --- | --- | --- | --- | --- | --- | --- | --- | --- | --- | --- | --- | --- | --- | --- | --- | --- | --- | --- | --- | --- | --- | --- | --- | --- | --- | --- | --- | --- | --- | --- | --- | --- | --- | --- | --- | --- | --- | --- | --- | --- | --- | --- | --- | --- | --- | --- | --- | --- | --- | --- | --- | --- | --- | --- | --- | --- | --- | --- | --- | --- | --- | --- | --- | --- | --- | --- | --- | --- | --- | --- | --- | --- | --- | --- | --- | --- | --- | --- | --- | --- | --- | --- | --- | --- | --- | --- | --- | --- | --- | --- | --- | --- | --- | --- | --- | --- | --- | --- | --- | --- | --- | --- | --- | --- | --- | --- | --- | --- | --- | --- | --- | --- | --- | --- | --- | --- | --- | --- | --- | --- | --- | --- | --- | --- | --- | --- | --- | --- | --- | --- | --- | --- | --- | --- | --- | --- | --- | --- | --- | --- | --- | --- | --- | --- | --- | --- | --- | --- | --- | --- | --- | --- | --- | --- | --- | --- | --- | --- | --- | --- | --- | --- | --- | --- | --- | --- | --- | --- | --- | --- | --- | --- | --- | --- | --- | --- | --- | --- | --- | --- | --- | --- | --- | --- | --- | --- | --- | --- | --- | --- | --- | --- | --- | --- | --- | --- | --- | --- | --- | --- | --- | --- | --- | --- | --- | --- | --- | --- | --- | --- | --- | --- | --- | --- | --- | --- | --- | --- | --- | --- | --- | --- | --- | --- | --- | --- | --- | --- | --- | --- | --- | --- | --- | --- | --- | --- | --- | --- | --- | --- | --- | --- |
|  |
| | **Table S4.** List of genes related to ‘Transcription’ from the MElightcyan module | | | | --- | --- | --- | | **ID** | **KOG_class_annotation** | **NR_annotation** | | MD00G1000500 | Transcription | PREDICTED: myb family transcription factor PHL11 [*Pyrus* x *bretschneideri*] | | MD00G1017500 | Transcription | PREDICTED: LOW QUALITY PROTEIN: zinc finger protein CONSTANS-LIKE 5-like [*Malus* *domestica*] | | MD00G1032600 | Transcription | MYB24 [*Malus* *domestica*] | | MD00G1104700 | Transcription | protein ODORANT1-like [*Malus* *domestica*] | | MD00G1115600 | Transcription | PREDICTED: B3 domain-containing transcription factor LEC2-like [*Malus* *domestica*] | | MD00G1169600 | Transcription | PREDICTED: transcription factor MYB1R1-like [*Malus* *domestica*] | | MD00G1206800 | Transcription | PREDICTED: SWI/SNF complex subunit SWI3B-like [*Malus* *domestica*] | | MD01G1036200 | Transcription | PREDICTED: homeobox-leucine zipper protein ATHB-6-like isoform X1 [*Malus* *domestica*] | | MD01G1060200 | Transcription | PREDICTED: transcription factor EGL1-like [*Pyrus* x *bretschneideri*] | | MD01G1082700 | Transcription | PREDICTED: transcription factor bHLH13-like [*Malus* *domestica*] | | MD01G1083000 | Transcription | PREDICTED: ethylene-responsive transcription factor ERF023-like [*Malus* *domestica*] | | MD01G1083400 | Transcription | PREDICTED: auxin response factor 18-like isoform X1 [*Malus* *domestica*] | | MD01G1084400 | Transcription | PREDICTED: myb-related protein 308-like [*Malus* *domestica*] | | MD01G1084700 | Transcription | PREDICTED: dof zinc finger protein DOF2.5-like isoform X1 [*Malus* *domestica*] | | MD01G1100800 | Transcription | PREDICTED: protein RNA-directed DNA methylation 3-like isoform X1 [*Pyrus* x *bretschneideri*] | | MD01G1108000 | Transcription | PREDICTED: GATA transcription factor 8-like [*Malus* *domestica*] | | MD01G1114600 | Transcription | PREDICTED: elongator complex protein 6-like [*Malus* *domestica*] | | MD01G1155800 | Transcription | PREDICTED: trihelix transcription factor ASIL2-like [*Malus* *domestica*] | | MD01G1158600 | Transcription | DREB2 [*Malus* sieversii] | | MD01G1177000 | Transcription | ethylene-responsive transcription factor 2-like [*Malus* *domestica*] | | MD01G1191600 | Transcription | PREDICTED: transcription factor bHLH120-like isoform X1 [*Malus* *domestica*] | | MD01G1198700 | Transcription | heat shock factor protein 4 [*Malus* *domestica*] | | MD02G1030900 | Transcription | ethylene-responsive transcription factor WIN1-like [*Malus* *domestica*] | | MD02G1039600 | Transcription | PREDICTED: LOW QUALITY PROTEIN: DELLA protein GAI [*Malus* *domestica*] | | MD02G1046900 | Transcription | heat stress transcription factor C-1 [*Malus* *domestica*] | | MD02G1064400 | Transcription | PREDICTED: probable RNA-dependent RNA polymerase 3 [*Malus* *domestica*] | | MD02G1082000 | Transcription | PREDICTED: heat stress transcription factor A-2-like [*Malus* *domestica*] | | MD02G1087900 | Transcription | PREDICTED: transcription factor TT2 [*Pyrus* x *bretschneideri*] | | MD02G1125900 | Transcription | RNA polymerase II largest subunit, partial [Psilotum nudum] | | MD02G1136100 | Transcription | PREDICTED: GATA transcription factor 5 [*Malus* *domestica*] | | MD02G1156800 | Transcription | BEL1-like homeodomain protein 2 [*Malus* *domestica*] | | MD02G1171300 | Transcription | PREDICTED: ethylene-responsive transcription factor ERF109 [*Malus* *domestica*] | | MD02G1176200 | Transcription | PREDICTED: ethylene-responsive transcription factor ERF010 [*Malus* *domestica*] | | MD02G1179000 | Transcription | PREDICTED: transcription factor MYB44-like [*Malus* *domestica*] | | MD02G1189300 | Transcription | PREDICTED: basic leucine zipper 8-like [*Malus* *domestica*] | | MD02G1189500 | Transcription | PREDICTED: growth-regulating factor 1 isoform X1 [*Malus* *domestica*] | | MD02G1190000 | Transcription | PREDICTED: AP2-like ethylene-responsive transcription factor AIL1 isoform X4 [*Malus* *domestica*] | | MD02G1192800 | Transcription | homeobox-leucine zipper protein HAT22-like [*Malus* *domestica*] | | MD02G1259300 | Transcription | PREDICTED: transcription factor DIVARICATA-like [*Malus* *domestica*] | | MD02G1263900 | Transcription | PREDICTED: LOW QUALITY PROTEIN: protein AATF-like [*Malus* *domestica*] | | MD02G1265300 | Transcription | PREDICTED: ethylene-responsive transcription factor WRI1 [*Pyrus* x *bretschneideri*] | | MD02G1302300 | Transcription | PREDICTED: RNA polymerase sigma factor sigD, chloroplastic [*Pyrus* x *bretschneideri*] | | MD03G1076200 | Transcription | PREDICTED: transcription factor DIVARICATA-like [*Malus* *domestica*] | | MD03G1107900 | Transcription | PREDICTED: ethylene-responsive transcription factor RAP2-7-like isoform X1 [*Malus* *domestica*] | | MD03G1118500 | Transcription | PREDICTED: homeobox-leucine zipper protein REVOLUTA-like [*Malus* *domestica*] | | MD03G1122400 | Transcription | PREDICTED: BEL1-like homeodomain protein 9 [*Pyrus* x *bretschneideri*] | | MD03G1222700 | Transcription | PREDICTED: NAC domain-containing protein 72-like [*Malus* *domestica*] | | MD03G1231800 | Transcription | ethylene-responsive transcription factor 4-like [*Malus* *domestica*] | | MD03G1239100 | Transcription | MdTCP4B [*Malus* *domestica*] | | MD03G1258300 | Transcription | PREDICTED: heat stress transcription factor A-6b [*Pyrus* x *bretschneideri*] | | MD03G1266300 | Transcription | PREDICTED: basic helix-loop-helix protein A-like [*Malus* *domestica*] | | MD04G1023500 | Transcription | PREDICTED: transcription factor bHLH77 [*Malus* *domestica*] | | MD04G1055300 | Transcription | PREDICTED: trihelix transcription factor ASIL2-like [*Malus* *domestica*] | | MD04G1058000 | Transcription | PREDICTED: ethylene-responsive transcription factor 2-like [*Malus* *domestica*] | | MD04G1058200 | Transcription | PREDICTED: ethylene-responsive transcription factor 5-like [*Malus* *domestica*] | | MD04G1061200 | Transcription | PREDICTED: homeobox-leucine zipper protein HAT4-like [*Malus* *domestica*] | | MD04G1067800 | Transcription | DREB1b transcription factor [*Malus* sieversii] | | MD04G1069300 | Transcription | PREDICTED: transcription factor TCP9-like [*Pyrus* x *bretschneideri*] | | MD04G1083800 | Transcription | PREDICTED: axial regulator YABBY 1-like isoform X1 [*Malus* *domestica*] | | MD04G1129500 | Transcription | MYB91 [*Malus* *domestica*] | | MD04G1147800 | Transcription | PREDICTED: transcription factor DIVARICATA [*Malus* *domestica*] | | MD04G1155800 | Transcription | PREDICTED: transcription factor TGA4-like [*Malus* *domestica*] | | MD04G1184900 | Transcription | MYB domain class transcription factor [*Malus* *domestica*] | | MD04G1224700 | Transcription | PREDICTED: transcription factor UNE12-like isoform X2 [*Malus* *domestica*] | | MD05G1024500 | Transcription | PREDICTED: trihelix transcription factor GT-2-like [*Pyrus* x *bretschneideri*] | | MD05G1072500 | Transcription | PREDICTED: B3 domain-containing transcription factor VRN1-like isoform X1 [*Malus* *domestica*] | | MD05G1074600 | Transcription | PREDICTED: myb family transcription factor EFM-like [*Pyrus* x *bretschneideri*] | | MD05G1082000 | Transcription | PREDICTED: ABSCISIC ACID-INSENSITIVE 5-like protein 5 [*Malus* *domestica*] | | MD05G1089000 | Transcription | PREDICTED: glycine-rich protein 2-like [*Malus* *domestica*] | | MD05G1089600 | Transcription | transcription factor MYB44-like [*Malus* *domestica*] | | MD05G1120600 | Transcription | PREDICTED: myb-related protein 308 [*Malus* *domestica*] | | MD05G1171400 | Transcription | PREDICTED: ethylene-responsive transcription factor-like protein At4g13040 [*Malus* *domestica*] | | MD05G1182300 | Transcription | PREDICTED: transcription factor PIF1-like isoform X1 [*Malus* *domestica*] | | MD05G1205900 | Transcription | auxin-induced protein 22D-like [*Malus* *domestica*] | | MD05G1222600 | Transcription | PREDICTED: cyclin-T1-3-like [*Malus* *domestica*] | | MD05G1229500 | Transcription | PREDICTED: DELLA protein RGL1-like [*Malus* *domestica*] | | MD05G1238400 | Transcription | PREDICTED: myb family transcription factor APL-like [*Malus* *domestica*] | | MD05G1255100 | Transcription | PREDICTED: transcription initiation factor TFIID subunit 8-like [*Pyrus* x *bretschneideri*] | | MD05G1279200 | Transcription | auxin response factor 6-like [*Malus* *domestica*] | | MD05G1293700 | Transcription | PREDICTED: floral homeotic protein AGAMOUS-like isoform X1 [*Malus* *domestica*] | | MD05G1309400 | Transcription | PREDICTED: auxin response factor 3-like [*Malus* *domestica*] | | MD05G1311600 | Transcription | PREDICTED: ethylene-responsive transcription factor 12-like [*Malus* *domestica*] | | MD05G1336600 | Transcription | PREDICTED: transcription factor ILR3-like [*Malus* *domestica*] | | MD06G1034300 | Transcription | transcription factor MYC2 [*Malus* *domestica*] | | MD06G1051900 | Transcription | PREDICTED: ethylene-responsive transcription factor 5-like [*Pyrus* x *bretschneideri*] | | MD06G1054800 | Transcription | PREDICTED: homeobox-leucine zipper protein HAT4-like [*Malus* *domestica*] | | MD06G1070100 | Transcription | PREDICTED: LOW QUALITY PROTEIN: transcription factor TCP9-like [*Malus* *domestica*] | | MD06G1072200 | Transcription | PREDICTED: dehydration-responsive element-binding protein 1E-like [*Malus* *domestica*] | | MD06G1072300 | Transcription | CBF3 [*Malus* sieversii] | | MD06G1125700 | Transcription | PREDICTED: dehydration-responsive element-binding protein 1E-like [*Malus* *domestica*] | | MD06G1135700 | Transcription | PREDICTED: NAC domain-containing protein 83-like [*Malus* *domestica*] | | MD06G1140100 | Transcription | PREDICTED: truncated transcription factor CAULIFLOWER A-like isoform X3 [*Malus* *domestica*] | | MD06G1141200 | Transcription | PREDICTED: nuclear transcription factor Y subunit C-1-like [*Malus* *domestica*] | | MD06G1155100 | Transcription | PREDICTED: elongator complex protein 3-like [*Pyrus* x *bretschneideri*] | | MD06G1167200 | Transcription | PREDICTED: transcription factor MYB86-like [*Malus* *domestica*] | | MD06G1182400 | Transcription | PREDICTED: two-component response regulator ARR5-like [*Malus* *domestica*] | | MD06G1191800 | Transcription | PREDICTED: transcription factor TCP14-like [*Malus* *domestica*] | | MD06G1206200 | Transcription | PREDICTED: transcription factor BIM1-like isoform X1 [*Malus* *domestica*] | | MD06G1217200 | Transcription | PREDICTED: transcription factor WER [*Malus* *domestica*] | | MD07G1099400 | Transcription | PREDICTED: ethylene-responsive transcription factor ERF034-like [*Malus* *domestica*] | | MD07G1104900 | Transcription | PREDICTED: ethylene-responsive transcription factor 2-like [*Malus* *domestica*] | | MD07G1111600 | Transcription | PREDICTED: squamosa promoter-binding-like protein 7 isoform X1 [*Malus* *domestica*] | | MD07G1112200 | Transcription | GATA transcription factor 4-like [*Malus* *domestica*] | | MD07G1113200 | Transcription | PREDICTED: transcription factor UNE10 isoform X1 [*Malus* *domestica*] | | MD07G1117200 | Transcription | axial regulator YABBY 1-like [*Malus* *domestica*] | | MD07G1151000 | Transcription | PREDICTED: transcription factor bHLH13 [*Malus* *domestica*] | | MD07G1151700 | Transcription | PREDICTED: ethylene-responsive transcription factor ERF023-like [*Malus* *domestica*] | | MD07G1153200 | Transcription | PREDICTED: myb-related protein 308-like [*Pyrus* x *bretschneideri*] | | MD07G1160700 | Transcription | PREDICTED: transcription factor bHLH57-like isoform X1 [*Malus* *domestica*] | | MD07G1173900 | Transcription | PREDICTED: GATA transcription factor 8-like [*Malus* *domestica*] | | MD07G1174000 | Transcription | PREDICTED: auxin response factor 18-like [*Malus* *domestica*] | | MD07G1182300 | Transcription | PREDICTED: two-component response regulator-like PRR73 [*Malus* *domestica*] | | MD07G1205600 | Transcription | PREDICTED: BEL1-like homeodomain protein 9 [*Malus* *domestica*] | | MD07G1211300 | Transcription | PREDICTED: myb-related protein 306-like [*Malus* *domestica*] | | MD07G1248600 | Transcription | ethylene-responsive transcription factor ERF105 [*Malus* *domestica*] | | MD07G1266500 | Transcription | PREDICTED: heat stress transcription factor B-2a-like [*Malus* *domestica*] | | MD07G1285600 | Transcription | PREDICTED: RNA polymerase sigma factor sigE, chloroplastic/mitochondrial-like isoform X1 [*Pyrus* x *bretschneideri*] | | MD07G1311500 | Transcription | PREDICTED: RNA polymerase sigma factor sigA-like isoform X1 [*Malus* *domestica*] | | MD08G1015500 | Transcription | PREDICTED: auxin response factor 5-like [*Malus* *domestica*] | | MD08G1030400 | Transcription | PREDICTED: protein RADIALIS-like 3 [*Pyrus* x *bretschneideri*] | | MD08G1040100 | Transcription | PREDICTED: dof zinc finger protein DOF3.4-like [*Malus* *domestica*] | | MD08G1049600 | Transcription | zinc finger protein CONSTANS-LIKE 4-like [*Malus* *domestica*] | | MD08G1070800 | Transcription | PREDICTED: transcription repressor MYB6-like [*Malus* *domestica*] | | MD08G1092000 | Transcription | transcription factor MYB44-like [*Malus* *domestica*] | | MD08G1101500 | Transcription | PREDICTED: thioredoxin-like 4, chloroplastic [*Pyrus* x *bretschneideri*] | | MD08G1107400 | Transcription | PREDICTED: transcriptional activator Myb-like [*Malus* *domestica*] | | MD08G1112900 | Transcription | homeobox-leucine zipper protein ATHB-8 [*Malus* *domestica*] | | MD08G1113200 | Transcription | GATA transcription factor 12-like [*Malus* *domestica*] | | MD08G1114100 | Transcription | ethylene-responsive transcription factor TINY-like [*Malus* *domestica*] | | MD08G1123300 | Transcription | PREDICTED: basic leucine zipper 9-like [*Malus* *domestica*] | | MD08G1151300 | Transcription | auxin-responsive protein IAA29-like [*Malus* *domestica*] | | MD08G1180200 | Transcription | DREB6.1 [*Malus* sieversii] | | MD08G1188500 | Transcription | PREDICTED: homeobox-leucine zipper protein ATHB-6-like [*Malus* *domestica*] | | MD08G1190500 | Transcription | transcription factor bHLH96-like [*Malus* *domestica*] | | MD08G1207700 | Transcription | PREDICTED: transcription factor bHLH61-like [*Malus* *domestica*] | | MD08G1220500 | Transcription | PREDICTED: transcription repressor MYB6-like [*Malus* *domestica*] | | MD08G1224700 | Transcription | PREDICTED: G-box-binding factor 4-like [*Malus* *domestica*] | | MD09G1003800 | Transcription | PREDICTED: transcription factor ICE1 [*Malus* *domestica*] | | MD09G1009100 | Transcription | PREDICTED: truncated transcription factor CAULIFLOWER D-like isoform X3 [*Pyrus* x *bretschneideri*] | | MD09G1019800 | Transcription | PREDICTED: zinc finger protein CONSTANS-LIKE 6-like [*Pyrus* x *bretschneideri*] | | MD09G1049000 | Transcription | PREDICTED: homeobox-leucine zipper protein HAT7-like [*Malus* *domestica*] | | MD09G1049300 | Transcription | PREDICTED: transcription factor PRE1 [*Malus* *domestica*] | | MD09G1073900 | Transcription | MADS domain class transcription factor [*Malus* *domestica*] | | MD09G1079200 | Transcription | PREDICTED: zinc finger protein CONSTANS-LIKE 2 [*Malus* *domestica*] | | MD09G1121700 | Transcription | PREDICTED: scarecrow-like transcription factor PAT1 [*Malus* *domestica*] | | MD09G1146000 | Transcription | PREDICTED: transcription factor PIF4-like [*Malus* *domestica*] | | MD09G1150700 | Transcription | PREDICTED: probable WRKY transcription factor 71 [*Pyrus* x *bretschneideri*] | | MD09G1155200 | Transcription | agamous-like MADS-box protein AGL1 [*Malus* *domestica*] | | MD09G1183800 | Transcription | PREDICTED: transcription factor WER-like [*Malus* *domestica*] | | MD09G1184000 | Transcription | uncharacterized protein LOC103453608 [*Malus* *domestica*] | | MD09G1202300 | Transcription | auxin-responsive protein IAA26-like [*Malus* *domestica*] | | MD09G1206700 | Transcription | ethylene-responsive transcription factor TINY-like [*Malus* *domestica*] | | MD09G1232700 | Transcription | PREDICTED: transcription factor TCP4-like [*Malus* *domestica*] | | MD09G1261200 | Transcription | myb-related protein 308-like [*Malus* *domestica*] | | MD09G1279300 | Transcription | PREDICTED: protein REVEILLE 1-like [*Malus* *domestica*] | | MD09G1282200 | Transcription | PREDICTED: auxin response factor 8-like [*Malus* *domestica*] | | MD09G1292300 | Transcription | PREDICTED: protein RADIALIS-like 6 [*Pyrus* x *bretschneideri*] | | MD10G1025100 | Transcription | PREDICTED: trihelix transcription factor GT-2-like [*Malus* *domestica*] | | MD10G1057000 | Transcription | PREDICTED: transcription factor GTE7-like [*Malus* *domestica*] | | MD10G1068000 | Transcription | PREDICTED: BEL1-like homeodomain protein 6 [*Malus* *domestica*] | | MD10G1098900 | Transcription | PREDICTED: transcription factor bHLH113-like [*Malus* *domestica*] | | MD10G1115500 | Transcription | PREDICTED: transcription factor bHLH18-like [*Pyrus* x *bretschneideri*] | | MD10G1151700 | Transcription | PREDICTED: transcription factor IBH1-like [*Malus* *domestica*] | | MD10G1170600 | Transcription | PREDICTED: transcription factor PIF1 isoform X1 [*Malus* *domestica*] | | MD10G1191300 | Transcription | PREDICTED: ethylene-responsive transcription factor 8-like [*Malus* *domestica*] | | MD10G1192900 | Transcription | PREDICTED: auxin-induced protein AUX28 [*Malus* *domestica*] | | MD10G1193000 | Transcription | uncharacterized protein LOC103445716 [*Malus* *domestica*] | | MD10G1235200 | Transcription | PREDICTED: transcription initiation factor TFIID subunit 8 [*Malus* *domestica*] | | MD10G1257900 | Transcription | PREDICTED: auxin response factor 6 [*Malus* *domestica*] | | MD10G1271000 | Transcription | PREDICTED: floral homeotic protein AGAMOUS isoform X1 [*Malus* *domestica*] | | MD10G1290900 | Transcription | PREDICTED: ethylene-responsive transcription factor 12-like [*Malus* *domestica*] | | MD10G1291800 | Transcription | PREDICTED: squamosa promoter-binding-like protein 3 [*Malus* *domestica*] | | MD10G1306300 | Transcription | PREDICTED: MADS-box protein AGL24-like [*Malus* *domestica*] | | MD10G1324500 | Transcription | PREDICTED: probable WRKY transcription factor 31 [*Malus* *domestica*] | | MD10G1326500 | Transcription | PREDICTED: homeobox protein knotted-1-like LET6 isoform X2 [*Malus* *domestica*] | | MD11G1007500 | Transcription | PREDICTED: transcription repressor MYB5-like [*Malus* *domestica*] | | MD11G1028100 | Transcription | PREDICTED: transcription factor bHLH130-like isoform X2 [*Malus* *domestica*] | | MD11G1136800 | Transcription | homeobox-leucine zipper protein REVOLUTA-like [*Malus* *domestica*] | | MD11G1140600 | Transcription | PREDICTED: BEL1-like homeodomain protein 9 isoform X2 [*Malus* *domestica*] | | MD11G1196300 | Transcription | PREDICTED: scarecrow-like protein 3 [*Malus* *domestica*] | | MD11G1251800 | Transcription | SPL domain class transcription factor [*Malus* *domestica*] | | MD11G1252800 | Transcription | ethylene-responsive transcription factor 4-like [*Malus* *domestica*] | | MD11G1258900 | Transcription | PREDICTED: transcription factor TCP4 [*Malus* *domestica*] | | MD11G1286900 | Transcription | BHLH domain class transcription factor [*Malus* *domestica*] | | MD11G1306500 | Transcription | ethylene-responsive transcription factor ERF071-like [*Malus* *domestica*] | | MD12G1018900 | Transcription | PREDICTED: trihelix transcription factor PTL [*Malus* *domestica*] | | MD12G1034100 | Transcription | PREDICTED: probable CCR4-associated factor 1 homolog 11 [*Malus* *domestica*] | | MD12G1060200 | Transcription | SBP-box transcription factor [*Malus* *domestica*] | | MD12G1063900 | Transcription | PREDICTED: transcription factor bHLH48-like [*Malus* *domestica*] | | MD12G1111400 | Transcription | PREDICTED: uncharacterized protein LOC103444807 isoform X3 [*Malus* *domestica*] | | MD12G1115100 | Transcription | PREDICTED: transcription factor TCP11 [*Malus* *domestica*] | | MD12G1125800 | Transcription | PREDICTED: LOW QUALITY PROTEIN: uncharacterized protein LOC103423389 [*Malus* *domestica*] | | MD12G1126100 | Transcription | PREDICTED: protein REVEILLE 8 isoform X1 [*Malus* *domestica*] | | MD12G1142500 | Transcription | PREDICTED: transcription factor AS1-like [*Malus* *domestica*] | | MD12G1168300 | Transcription | PREDICTED: trihelix transcription factor GT-3b [*Malus* *domestica*] | | MD12G1196300 | Transcription | PREDICTED: protein SCARECROW 2-like [*Malus* *domestica*] | | MD12G1198900 | Transcription | transcription factor PIF3-like [*Malus* *domestica*] | | MD12G1205700 | Transcription | PREDICTED: homeobox protein knotted-1-like 1 isoform X1 [*Malus* *domestica*] | | MD12G1238000 | Transcription | PREDICTED: trihelix transcription factor GTL2-like [*Malus* *domestica*] | | MD12G1244500 | Transcription | PREDICTED: GRF1-interacting factor 1-like [*Malus* *domestica*] | | MD12G1246500 | Transcription | cell division cycle 5-like protein isoform X1 [Prunus avium] | | MD13G1022100 | Transcription | spur-type DELLA protein [*Malus* *domestica*] | | MD13G1035200 | Transcription | PREDICTED: auxin-responsive protein IAA32 [*Pyrus* x *bretschneideri*] | | MD13G1039900 | Transcription | PREDICTED: myb-related protein 305 [*Malus* *domestica*] | | MD13G1041300 | Transcription | PREDICTED: probable RNA-dependent RNA polymerase 1 [*Malus* *domestica*] | | MD13G1042200 | Transcription | PREDICTED: zinc finger protein CONSTANS-LIKE 6-like [*Malus* *domestica*] | | MD13G1046100 | Transcription | PREDICTED: AP2/ERF and B3 domain-containing transcription factor RAV1-like [*Malus* *domestica*] | | MD13G1059200 | Transcription | truncated transcription factor CAULIFLOWER A [*Malus* *domestica*] | | MD13G1064700 | Transcription | PREDICTED: probable WRKY transcription factor 57 isoform X1 [*Malus* *domestica*] | | MD13G1075000 | Transcription | PREDICTED: transcription factor MYB51 isoform X1 [*Malus* *domestica*] | | MD13G1083200 | Transcription | PREDICTED: transcription factor bHLH79-like [*Malus* *domestica*] | | MD13G1084100 | Transcription | transcription factor MYB1R1-like [*Malus* *domestica*] | | MD13G1120300 | Transcription | PREDICTED: squamosa promoter-binding-like protein 2 [*Malus* *domestica*] | | MD13G1122900 | Transcription | PREDICTED: transcription factor TCP20-like [*Malus* *domestica*] | | MD13G1159000 | Transcription | PREDICTED: GATA transcription factor 15 [*Pyrus* x *bretschneideri*] | | MD13G1222200 | Transcription | ARF domain class transcription factor [*Malus* *domestica*] | | MD13G1238400 | Transcription | transcription factor TCP7-like [*Malus* *domestica*] | | MD13G1239700 | Transcription | PREDICTED: transcription factor bHLH35-like [*Malus* *domestica*] | | MD13G1263800 | Transcription | PREDICTED: zinc finger protein CONSTANS-LIKE 5-like [*Malus* *domestica*] | | MD14G1001600 | Transcription | PREDICTED: uncharacterized protein LOC103424216 [*Malus* *domestica*] | | MD14G1010400 | Transcription | PREDICTED: WUSCHEL-related homeobox 11-like [*Malus* *domestica*] | | MD14G1016900 | Transcription | PREDICTED: trihelix transcription factor PTL-like [*Malus* *domestica*] | | MD14G1018400 | Transcription | PREDICTED: protein BUD31 homolog 1-like [*Malus* *domestica*] | | MD14G1023000 | Transcription | PREDICTED: glucosamine 6-phosphate N-acetyltransferase-like [*Malus* *domestica*] | | MD14G1060200 | Transcription | PREDICTED: squamosa promoter-binding-like protein 9 [*Malus* *domestica*] | | MD14G1064200 | Transcription | PREDICTED: transcription factor bHLH48-like [*Malus* *domestica*] | | MD14G1100900 | Transcription | PREDICTED: RNA polymerase sigma factor sigB isoform X2 [*Malus* *domestica*] | | MD14G1120000 | Transcription | PREDICTED: ethylene-responsive transcription factor CRF2-like [*Pyrus* x *bretschneideri*] | | MD14G1127700 | Transcription | PREDICTED: ethylene-responsive transcription factor ERF061 [*Malus* *domestica*] | | MD14G1162200 | Transcription | PREDICTED: transcription factor GTE10 [*Malus* *domestica*] | | MD14G1172900 | Transcription | PREDICTED: transcription factor MYB86-like [*Pyrus* x *bretschneideri*] | | MD14G1180900 | Transcription | PREDICTED: transcription factor WER-like [*Malus* *domestica*] | | MD14G1188400 | Transcription | PREDICTED: two-component response regulator ARR5-like [*Malus* *domestica*] | | MD14G1198200 | Transcription | transcription factor TCP14 [*Malus* *domestica*] | | MD14G1222200 | Transcription | PREDICTED: transcription factor DIVARICATA-like [*Pyrus* x *bretschneideri*] | | MD14G1226400 | Transcription | PREDICTED: zinc finger protein CONSTANS-LIKE 6-like [*Pyrus* x *bretschneideri*] | | MD14G1234600 | Transcription | myb-related protein 308-like [*Malus* *domestica*] | | MD15G1025600 | Transcription | PREDICTED: protein RADIALIS-like 6 [*Malus* *domestica*] | | MD15G1038900 | Transcription | PREDICTED: transcription initiation factor TFIID subunit 5-like [*Malus* *domestica*] | | MD15G1050300 | Transcription | PREDICTED: transcription factor MYB114-like [*Malus* *domestica*] | | MD15G1051400 | Transcription | PREDICTED: anthocyanin regulatory C1 protein-like [*Malus* *domestica*] | | MD15G1064500 | Transcription | PREDICTED: transcription factor bHLH74 isoform X1 [*Malus* *domestica*] | | MD15G1078200 | Transcription | PREDICTED: probable WRKY transcription factor 7 [*Malus* *domestica*] | | MD15G1082700 | Transcription | PREDICTED: thioredoxin-like 4, chloroplastic [*Malus* *domestica*] | | MD15G1102300 | Transcription | PREDICTED: basic leucine zipper 9-like [*Malus* *domestica*] | | MD15G1154600 | Transcription | PREDICTED: transcription factor bHLH66-like [*Pyrus* x *bretschneideri*] | | MD15G1185400 | Transcription | PREDICTED: heat stress transcription factor C-1-like [*Malus* *domestica*] | | MD15G1191400 | Transcription | PREDICTED: transcription factor bHLH68-like isoform X2 [*Malus* *domestica*] | | MD15G1191800 | Transcription | PREDICTED: auxin-responsive protein IAA27-like [*Pyrus* x *bretschneideri*] | | MD15G1213400 | Transcription | PREDICTED: nuclear transcription factor Y subunit A-4-like [*Malus* *domestica*] | | MD15G1221400 | Transcription | PREDICTED: auxin response factor 19-like isoform X2 [*Malus* *domestica*] | | MD15G1249700 | Transcription | GATA transcription factor 5-like [*Malus* *domestica*] | | MD15G1263000 | Transcription | PREDICTED: scarecrow-like protein 4 [*Malus* *domestica*] | | MD15G1266500 | Transcription | PREDICTED: LOW QUALITY PROTEIN: scarecrow-like protein 15 [*Malus* *domestica*] | | MD15G1275300 | Transcription | PREDICTED: dof zinc finger protein DOF3.4-like [*Malus* *domestica*] | | MD15G1285600 | Transcription | PREDICTED: transcription factor SPATULA [*Malus* *domestica*] | | MD15G1300200 | Transcription | PREDICTED: bZIP transcription factor 53-like [*Malus* *domestica*] | | MD15G1302900 | Transcription | PREDICTED: homeobox-leucine zipper protein HAT22-like [*Pyrus* x *bretschneideri*] | | MD15G1306400 | Transcription | PREDICTED: transcription factor bHLH93-like isoform X1 [*Malus* *domestica*] | | MD15G1365500 | Transcription | DREB6.2 [*Malus* sieversii] | | MD15G1377800 | Transcription | BHLH domain class transcription factor [*Malus* *domestica*] | | MD15G1384600 | Transcription | PREDICTED: MADS-box protein SVP-like [*Malus* *domestica*] | | MD15G1396900 | Transcription | PREDICTED: ethylene-responsive transcription factor ERF021 [*Malus* *domestica*] | | MD15G1400400 | Transcription | PREDICTED: agamous-like MADS-box protein AGL12 [*Malus* *domestica*] | | MD15G1431700 | Transcription | PREDICTED: transcription factor TCP8-like [*Malus* *domestica*] | | MD16G1017900 | Transcription | PREDICTED: two-component response regulator ORR21-like [*Malus* *domestica*] | | MD16G1029400 | Transcription | PREDICTED: myb-related protein 308-like [*Malus* *domestica*] | | MD16G1043000 | Transcription | PREDICTED: zinc finger protein CONSTANS-LIKE 6-like [*Malus* *domestica*] | | MD16G1058500 | Transcription | MADS-box protein [*Malus* *domestica*] | | MD16G1069500 | Transcription | NAC domain class transcription factor [*Malus* *domestica*] | | MD16G1074800 | Transcription | transcription factor TCP15-like [*Malus* *domestica*] | | MD16G1079400 | Transcription | PREDICTED: homeobox-leucine zipper protein HAT5-like [*Malus* *domestica*] | | MD16G1083300 | Transcription | PREDICTED: transcription factor MYB1R1 [*Malus* *domestica*] | | MD16G1093900 | Transcription | PREDICTED: transcription factor bHLH90 [*Malus* *domestica*] | | MD16G1120300 | Transcription | PREDICTED: squamosa promoter-binding-like protein 2 [*Malus* *domestica*] | | MD16G1121200 | Transcription | PREDICTED: calmodulin-binding transcription activator 4 [*Pyrus* x *bretschneideri*] | | MD16G1139000 | Transcription | PREDICTED: calmodulin-binding transcription activator 3-like isoform X1 [*Pyrus* x *bretschneideri*] | | MD16G1139100 | Transcription | PREDICTED: calmodulin-binding transcription activator 3-like isoform X2 [*Pyrus* x *bretschneideri*] | | MD16G1151400 | Transcription | PREDICTED: transcription factor bHLH147-like [*Malus* *domestica*] | | MD16G1206700 | Transcription | PREDICTED: auxin-responsive protein IAA14-like [*Malus* *domestica*] | | MD16G1234900 | Transcription | PREDICTED: GATA transcription factor 1-like [*Malus* *domestica*] | | MD16G1243300 | Transcription | PREDICTED: transcription factor TCP7 [*Pyrus* x *bretschneideri*] | | MD16G1248400 | Transcription | PREDICTED: protein CHLOROPLAST IMPORT APPARATUS 2-like isoform X1 [*Malus* *domestica*] | | MD16G1257700 | Transcription | PREDICTED: probable small nuclear ribonucleoprotein F [*Malus* *domestica*] | | MD16G1265000 | Transcription | PREDICTED: zinc finger protein CONSTANS-LIKE 5 isoform X1 [*Malus* *domestica*] | | MD16G1269900 | Transcription | PREDICTED: two-component response regulator-like APRR2 [*Malus* *domestica*] | | MD17G1020400 | Transcription | PREDICTED: zinc finger protein CONSTANS-LIKE 6 [*Malus* *domestica*] | | MD17G1035400 | Transcription | homeobox-leucine zipper protein HAT5 [*Malus* *domestica*] | | MD17G1049000 | Transcription | PREDICTED: homeobox-leucine zipper protein HAT7 [*Malus* *domestica*] | | MD17G1050900 | Transcription | PREDICTED: myb-related protein 306-like [*Malus* *domestica*] | | MD17G1069600 | Transcription | COL domain class transcription factor [*Malus* *domestica*] | | MD17G1073900 | Transcription | PREDICTED: LOW QUALITY PROTEIN: protein rough sheath 2 homolog [*Malus* *domestica*] | | MD17G1094200 | Transcription | PREDICTED: transcription factor CPC-like [*Malus* *domestica*] | | MD17G1128300 | Transcription | PREDICTED: scarecrow-like protein 23 [*Malus* *domestica*] | | MD17G1141300 | Transcription | PREDICTED: agamous-like MADS-box protein AGL1 isoform X1 [*Malus* *domestica*] | | MD17G1176100 | Transcription | PREDICTED: dof zinc finger protein DOF3.1-like [*Pyrus* x *bretschneideri*] | | MD17G1183500 | Transcription | PREDICTED: auxin-responsive protein IAA26-like [*Pyrus* x *bretschneideri*] | | MD17G1184100 | Transcription | MYBR domain class transcription factor [*Malus* *domestica*] | | MD17G1189100 | Transcription | PREDICTED: LOW QUALITY PROTEIN: auxin-responsive protein IAA21-like [*Malus* *domestica*] | | MD17G1190000 | Transcription | PREDICTED: transcription factor bHLH68-like [*Malus* *domestica*] | | MD17G1230100 | Transcription | heat stress transcription factor A-4b-like [*Malus* *domestica*] | | MD17G1234400 | Transcription | PREDICTED: transcription activator GLK2-like isoform X3 [*Pyrus* x *bretschneideri*] | | MD17G1260700 | Transcription | DELLA protein GAI-like [*Malus* *domestica*] | | MD17G1261500 | Transcription | PREDICTED: transcription repressor KAN1 isoform X2 [*Pyrus* x *bretschneideri*] | | MD17G1271400 | Transcription | PREDICTED: protein REVEILLE 1 [*Pyrus* x *bretschneideri*] | |
